# Role of Natural Stone Wastes and Minerals in the Alkali Activation Process: A Review

**DOI:** 10.3390/ma13102284

**Published:** 2020-05-15

**Authors:** Bartolomeo Coppola, Jean-Marc Tulliani, Paola Antonaci, Paola Palmero

**Affiliations:** 1 INSTM R.U. Lince Laboratory, Department of Applied Science and Technology, Politecnico di Torino, Corso Duca Degli Abruzzi, 24, 10129 Torino, Italy; bartolomeo.coppola@polito.it (B.C.); jeanmarc.tulliani@polito.it (J.-M.T.); paola.palmero@polito.it (P.P.); 2 Department of Structural, Geotechnical and Building Engineering, Politecnico di Torino, Corso Duca Degli Abruzzi, 24, 10129 Torino, Italy; 3 Responsible Risk Resilience Centre, Politecnico di Torino, Viale Mattioli 39, 10125 Torino, Italy

**Keywords:** alkali activation, geopolymer, stone waste, stone, minerals, alumino-silicate, carbonates, calcite, dolomite, reuse, sustainability

## Abstract

This review aims to provide a comprehensive assessment concerning alkali activation of natural stone wastes and minerals. In particular, the structure of the review is divided into two main sections in which the works dealing with alumino-silicate and carbonatic stones are discussed, respectively. Alumino-silicate stones are generally composed of quartz and feldspars, while carbonatic stones are mainly made of calcite and dolomite. The role of these minerals in the alkali activation process is discussed, attesting their influence in the development of the final product properties. In most of the works, authors use mineral additions only as fillers or aggregates and, in some cases, as a partial substitution of more traditional raw powders, such as metakaolin, fly ash, and granulated blast furnace slag. However, a few works in which alumino-silicate and carbonatic stone wastes are used as the main active components are discussed as well. Not only the raw materials, but also the entire alkali activation process and the curing conditions adopted in the literature studies here reviewed are systematically analyzed to improve the understanding of their effect on the physical, mechanical, and durability properties of the final products and to eventually foster the reuse of natural stone wastes for the purposes of sustainability in different applications.

## 1. Introduction

The interest towards geopolymers and alkali-activated materials arises due to the search for lower carbon dioxide (CO_2_) binders as compared to traditional cement [1,2,3,4,5].

The term ‘alkali-activated materials’ normally refers to aluminate-rich materials showing cementitious properties as a consequence of reactions initiated by an alkaline activator. However, as underlined by J. Davidovits [6], geopolymers and alkali-activated materials are characterized by significantly different structures, compositions, and properties. The former, in fact, are generated by polymer chemistry mechanisms (poly-sialate); the latter are just hydrated and/or precipitated products [7]. More in detail, geopolymer formation occurs via dissolution of silica (SiO_4_) and alumina (AlO_4_) tetrahedra and subsequent condensation phenomena, giving rise to larger alumino-silicate oligomers, which further condense to form large structural units [8]. In such a way, a three-dimensional polymer structure is formed, and this arrangement accounts for the high durability of geopolymers, which is generally superior to that of alkali-activated materials [6,7].

Traditionally, geopolymers are produced starting from metakaolin (obtained by the high-temperature calcination of kaolin [9]) as a source of Al and Si species, as well as an alkaline activator. The latter is a mixture of sodium or potassium hydroxide (NaOH or KOH) aqueous solution, added with liquid sodium silicate. Therefore, the geopolymer development requires the consumption of natural resources (both clay and the raw materials necessary to produce the chemical activators) and their high-energy treatment, so this process is associated with great environmental impacts [10,11,12].

The keys to reducing the carbon footprint associated with this process rely on the search for alternative raw materials and on the reduction of the amount and concentration of the alkaline activator, or at least of one of its components [11]. The most investigated strategy implies the use of specific industrial waste as an alternative alumina-silicate source, such as fly ash, ground granulated blast furnace slag, and various types of slags [10,12,13,14]. As a common feature, these industrial wastes are characterized not only by high silicate and aluminate contents, but also by their predominantly amorphous or poorly crystallized structure. This makes these materials highly reactive when exposed to the alkaline activator, thus favoring the dissolution and condensation reactions necessary to generate high-strength hardened materials.

**Figure 1:**
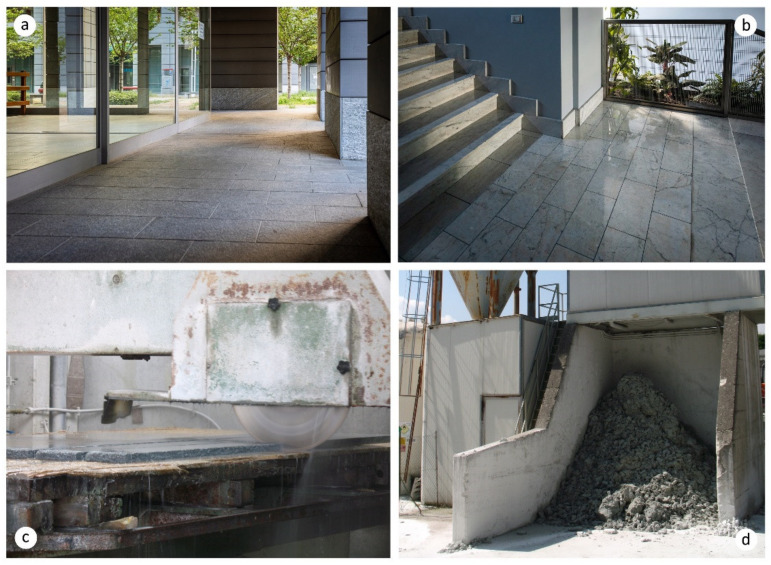
Typical installations of ornamental stones mainly containing alumino-silicate minerals (**a**) or carbonatic minerals (**b**); stone cutting process (**c**); stone waste powder resulting from the cutting process (**d**).

A more innovative approach implies the exploitation of industrial mineral wastes with highly crystalline structures, such as those produced during the extraction and processing of ornamental stones. In fact, huge volumes of natural stones are extracted worldwide, corresponding to impressive amounts of related wastes [15]. As an example, Figure 1 reports some pictures of typical installations of alumino-silicate- and carbonate-based ornamental stones, as well as pictures of the production process and resulting waste powders. With reference to marble and granite production, the world dimension is estimated to be approximatively 155 million tons (2014 data); China, India, Turkey, Iran, and Italy are the top five productive countries, which account for about 74% of the total world stone production dimension [16]. Moreover, this market, especially in Europe, is continuously increasing at an annual rate of 7% [17]. The stone manufacturing chain implies three main activities, here specified: (i) Extraction from quarry, (ii) finishing treatments (such as cutting, smoothing, and polishing), and (iii) transportation and sale [18]. These activities are associated with important landscape and environmental impacts, particularly during the extraction and cutting phases. During extraction, around 30% of the stone goes to scrap because of small size and/or irregular shape [19,20]. Moreover, large quantities of stone fines (named quarry dust) are generated, which could be harmful if dispersed in air, water, or soil [15]. On the other hand, the cutting and polishing phases produce a large amount of sludge, which is essentially constituted by a mix of quarry dusts and cooling water used in the working process [18]. It can be estimated that one ton of marble stone processed in a gang-saw or vertical/horizontal cutter produces almost one ton of slurry, 70% of which is water [20]. The water content is normally reduced by press filtration, thus producing a mud with a water content lower than 20% [21]. Generally, this waste is landfilled, while, in some cases, it is dumped directly into the ecosystem. Such improper disposal can be hazardous for the environment, producing soil and water contamination as well as necrotic conditions for flora and fauna. One concern is related to the fine particle size and the lack of pores, making the mud almost impermeable to oxygen and causing asphyxia in living organisms [22]. If a large amount of dust is dispersed in water, it can increase the water’s turbidity. As a consequence, light penetration is inhibited and photosynthesis activity is reduced, causing a lack of nutrients and modification of the food chain [23,24]. Finally, the dried mud and fine dust can be easily dragged by wind, becoming harmful to humans and animals through inhalation, inducing asthma, silicosis, and lung cancer [24]. A last issue concerns the possible presence of heavy metals in sludge due to cutting operations using frame saws. However, this concern is nowadays almost overcome thanks to the increasing use of metal-free diamond cutting discs.

In addition to the previously described health and environmental issues, quarry waste landfilling is very costly, corresponding to more than 3% of the operating costs of stone working plants [22]. Therefore, the incorporation of these wastes in other industrial processes could generate cost reduction and new business opportunities, while reducing the extraction of raw materials and preserving natural resources.

In light of this, the use of ornamental stone waste in the construction industry could be a smart solution to avoid landfilling and dust propagation in the environment. A recent work by Galetakis and Soultana [15] reviewed the use of quarry fines in the preparation of building materials. Authors found that slurries can be used as fine aggregates or as cement replacement [25,26,27], while solid waste can, in part, substitute aggregates in concrete mixtures, as already proposed in literature for other wastes as well [28,29,30,31,32].

More recently, the possibility of including fine quarry wastes into new alkali-activated products has been investigated, similarly to what other researchers are doing as regards alkali-activated materials containing mine tailings (i.e., the finely ground residue from ore extraction) [33,34,35,36]. The aim of this review, in fact, is to highlight the recent advances achieved by applying the alkaline activation approach to the finest fraction of the quarry wastes that have either a siliceous or carbonate nature. A summary of the papers discussed in this review is presented in Table 1. First, a classification based on the chemical nature of the waste is done. In the alumino-silicate class, a variety of mineral wastes are used, such as granite, albite, pietra serena, pumice, andesite, etc. The choice of alumino-silicate waste for the alkali activation process is based on the chemical similarity of these minerals to the commonly used raw materials, in which precursors rich in Si+Al and low in Ca contents give rise to inorganic polymers of sodium aluminum silicate hydrate (N-A-S-H) of high mechanical strength [37,38]. Concerning carbonates, the main exploited wastes derive from calcite, dolomite, marble, and limestone processing. Here, a possible role played by calcium on alkali-activated products is taken into consideration, since the formation of a calcium silicate hydrate (C-S-H) gel has been postulated in some previous works, with significant effects on the materials’ mechanical properties and durability [39,40,41,42,43,44]. It is interesting to observe from Table 1 that most publications deal with the use of mineral muds/dusts as additives to common geopolymer sources, such as metakaolin, fly ash, and granulated blast furnace slag. In fact, due to the highly crystalline nature of stone waste, its reactivity under alkaline conditions is significantly reduced, thus requiring an ‘active’ material to provide the setting and hardening stages and to control the whole process. However, very few studies rely on the exploitation of such very fine wastes as primary sources for alkali-activated materials [45,46]. To complete the overview of the raw materials, the chemical compositions of the alumino-silicate and carbonate materials used in the reviewed papers are reported in detail in Table 2 and Table 3, respectively.

Following the classification reported in Table 1, alumino-silicate and carbonate-containing alkali-activated materials are discussed in separate sections of this review (Section 2 and Section 3, respectively) and, for each of them, the roles of composition and processing (Section 2.1 and Section 3.1) as well as the properties of fresh and hardened materials (Section 2.2 and Section 3.2) are reviewed. Then, the specific role exerted by both mineral fines on the alkali activation process is considered (Section 2.2 and Section 3.2), followed by a final discussion (Section 4) aimed to provide a scientific contribution for maximizing the use of these wastes in industry and consequently reducing the impacts related to the manufacturing of ornamental stones.

## 2. Alkali-Activated Materials Based on Alumino-Silicate Minerals

### 2.1. Role of Raw Materials, Activators, and Curing Conditions

Different natural alumino-silicate minerals have been investigated in the scientific literature in combination with traditional alkali-activated materials. In particular, granite [46,47,48], albite [49], Pietra serena [50], Pisha sandstone [51,52], cordierite [53], and diatomite [54] have been used in combination with metakaolin (MK) [48,50,53] and fly ash (FA) [47,51]. As previously mentioned, only a few works investigated the use of alumino-silicate stones alone [46,49,51,52,54], i.e., without any other ‘active’ component. Alumino-silicate stones are mainly composed of quartz (SiO_2_) and feldspars, such as microcline (KAlSi_3_O_8_) and albite (NaAlSi_3_O_8_). In some cases, some phyllosilicates belonging to the mica group, like biotite and muscovite, can also be present. As regards the oxides, these materials are mainly composed of silica (with values ranging between 50% and 70%) and alumina (between 15% and 35%), as reported in Table 2. A certain difference is presented by diatomite, which is a naturally occurring siliceous sedimentary rock that is characterized by a very high silica amount (80.3%) and a lower alumina content (6.1%) [54].

Clausi et al. [50] investigated the possibility of using an ornamental stone, i.e., Pietra Serena (an Italian sandstone) in combination with MK for the production of mortars. MK/Pietra Serena alkali-activated mortars can be used in restoration of cultural heritage because they mimic the natural stone, thus allowing aesthetic compatibility [17]. Crushed Pietra Serena was used for aggregate preparation, keeping the clayey fraction as well, which provided the necessary color shades for aesthetic reasons.

Tchadjié et al. [48] used granite waste from Cameroon in the preparation of MK-based geopolymers. In particular, the authors fused granite powder (at 550 °C for 2 h) with Na_2_O at different weight percentages (from 10 to 60 wt.%, 10 wt.% of increments). Then, fused granite powder (d < 90 μm) was mixed with MK and activated with Na_2_SiO_3_ solution [48].

Hemra and Aungkavattana [53] investigated the influence of cordierite (Mg_2_Al_4_Si_2_O_18_) addition, from 0 to 50 wt.%, into MK-based geopolymers.

Hassan et al. [54] investigated the possibility of preparing geopolymers by mixing wood biomass ash (WBA) and diatomite. Even if diatomite is a sedimentary siliceous rock, it is mainly composed of alumino-silicates rich in amorphous iron.

Choi et al. [47] replaced fly ash (FA) and granulated blast furnace slag (GBFS) with a stone powder sludge derived from granite quarries. The authors investigated several replacement ratios (10, 20, and 30 wt.%, respectively) using the stone sludge as received, i.e., with a high water content (20.7%) [47].

An original contribution is provided by Palmero et al. [46], who exploited a granite quarry mud as a unique feedstock material for alkali-activated products, and this technology was the object of two recent patent applications [78,79]. Due to the almost fully crystalline nature of the raw powder, the developed material represents a clear innovation in the field of alkali-activation processes [46]. Similarly, Li et al. [51,52] investigated the alkali activation of Pisha sandstone (a Chinese sandstone) both alone [51,52] and mixed with FA [51]. Pisha sandstone is mainly composed of crystalline minerals, i.e., quartz and albite, with SiO_2_ and Al_2_O_3_, accounting for approximatively 80 wt.% of the whole composition (Table 2). Feng et al. [49] investigated the possibility of activating pure albite, but it was previously thermally treated with sodium hydroxide or sodium carbonate. In particular, albite was mixed with different amounts (10%, 30%, and 50% with respect to albite mass) of sodium hydroxide or sodium silicate and then heated at four different temperatures (850, 900, 1000, and 1150 °C, respectively) for 30 min [49].

Details related to curing conditions and alkaline solutions of the different reviewed papers are reported in Table 2. It can be observed that, in some researches, the materials were cured at 80 °C [46,47,51,52], even if in most of the works, curing at room temperature was preferred [48,49,50,52,53,54].

In addition, the heat-curing was always performed for a short time (a few hours or a few days), followed by a longer room temperature curing. For example, in the studies reported in [46,47], specimens were cured at 80 °C for 24 h and then kept at 23 °C until testing.

The curing atmosphere plays a role, too. For instance, Li et al. [51] investigated different curing conditions for geopolymers prepared using a Chinese sandstone (i.e., Pisha sandstone) by exposing the samples at 80 °C for 24 h, followed by room temperature curing performed under air or water. Authors reported lower mechanical properties in the case of water-cured samples, probably due to excessive water, which hindered the polycondensation reactions [51].

A further important parameter that affects the performance of the fresh and hardened materials is the activating solution. Li et al. investigated several formulations: In [51], the authors kept Na_2_SiO_3_ and water content constant while varying NaOH concentration, thus obtaining solutions at three different SiO_2_/Na_2_O molar ratios (M_s_), equal to 1.5, 2.0, and 3.0. They showed that the strength decreased by increasing M_s_. On the contrary, in [52], the same authors tested different activators (Na_2_SiO_3_, Na_2_CO_3_, Na_2_SO_4_, and NaOH, respectively) in order to investigate the role of pH on the development of alkali-activated materials. They showed that the higher the pH, the stronger the materials.

A recent and interesting approach deals with the design and development of one-part geopolymers, which are mixtures of solid alumino-silicate precursors and solid alkaline chemicals; the addition of ‘just water’ is responsible for the activation, similarly to cement technology [80,81,82]. The term is in opposition to the traditional two-part geopolymers, in which the solid precursors are mixed with a liquid alkaline solution: Issues related to the high viscosity and pH of the solution make the scalability of geopolymers difficult, restricting them to relatively small-scale applications and pre-cast components. Two previous researches investigated the possibility of preparing one-part geopolymers with natural minerals [49,54]. In particular, Feng et al. [49] prepared alkali-activated materials starting from a thermally treated albite and adding only water. The authors fixed the water/solid ratio at 0.30 and cured samples at room temperature in sealed containers [49]. On the other side, Hassan et al. [54] prepared one-part geopolymers mixing a dry activator with diatomite. In particular, the dry activator was prepared mixing a CaCO_3_-rich ash (WBA) with a dried solution of sodium hydroxide. Then, diatomite was mixed with different amounts of the dry activator and activated with water at a water/powder ratio of 0.27 and 3 wt.% of NaOH. Samples were finally cured at 23 °C and 99% relative humidity (RH).

### 2.2. Properties of Fresh and Hardened Materials

#### 2.2.1. Fresh Mixtures Properties

Properties of fresh mixtures are very important in alkali-activated materials not only for casting, but also for mechanical property development; in fact, water greatly influences workability and alkali activation reactions. In spite of this, only a few works studied the fresh properties of alkali-activated alumino-silicate mineral fines [46,49,54].

Palmero et al. [46] determined the apparent viscosity of alkali-activated granite pastes using a viscometer (in the range of 10–40 s^−1^) and found that values between 35 and 40 Pa·s were suitable for casting and mechanical property development.

Hassan et al. [54] studied initial and final setting times of one-part geopolymers made with a dry activator (WBA and NaOH) and diatomite. The authors measured an increase in setting time (both initial and final) with increasing dry activator content due to the dilution effect of calcium carbonate (contained in WBA) on the amount of amorphous reactive silica, and therefore on the formation of the binding phases [54]. Feng et al. [49] reported a setting time in the order of few minutes for one-part alkali-activated thermally treated albite. Indeed, this mixture containing alkalis (sodium hydroxide or sodium carbonate) was highly reactive, and a significant amount of heat was released after mixing with water [49].

#### 2.2.2. Mechanical Properties of Hardened Materials

The influence of alumino-silicate powder addition on the mechanical properties of the final alkali-activated material is reviewed in the following, and the highest compressive strengths achieved in the previous researches are summarized in Table 2.

Choi et al. [47] obtained two opposite trends in replacing FA and GBFS with granite sludge. In particular, for FA, at increasing sludge replacement, the compressive strength decreased, even at the lowest replacement (10 wt.% sludge). On the contrary, for GBFS, 10 wt.% replacement produced an increase of compressive strength (from 61.7 to 72.6 MPa), while higher substitutions provided lower values, but still higher than the reference neat GBFS material.

Clausi at al. [50] compared the mechanical properties of alkali-activated MK mortars prepared using a standard sand or Pietra Serena crushed sand as aggregates. The authors reported a significant decrease of both flexural and compressive strength compared to the reference mortar; however, the compressive strength achieved (21 MPa) was still suitable for masonry mortars. The strength decrease was attributed to the different particle shape (angular morphology) and to the higher presence of fines in Pietra Serena sand with respect to the standard sand.

Tchadjié et al. [48] investigated the mechanical properties of fused granite waste/MK mortars at different Na_2_O amounts in fused granite waste. In particular, the authors reported a progressive increase of compressive strength up to a Na_2_O content of 40 wt.% followed by a strength decrease. This Na_2_O wt.% was found to be the most favorable in terms of geopolymerization degree and strength development.

Hemra and Aungkavattana [53] obtained an increase of compressive strength at increasing cordierite addition in MK-based geopolymers. In particular, a 26% compressive strength increase (from 42.5 to 57.5 MPa) was measured by adding 30 wt.% of cordierite. The authors explained this result in terms of filler effect, because cordierite particles acted as aggregate and avoided crack propagation, improving the mechanical properties. Thus, no influence on the geopolymerization process was attributed to cordierite.

Li et al. [51,52] explored alkali activation of a Chinese sandstone (i.e., Pisha sandstone) both alone and mixed with FA, and investigated the effects of several parameters. First, for pure Pisha sandstone, the authors stated an important role of curing conditions; the 28 day compressive strength of samples cured under air (~6 MPa) is higher than that of water cured materials (~4 MPa) [51]. Second, the curing time showed a role too, since, in both cases, higher mechanical properties were achieved after 90 days of curing. Third, the higher the NaOH content in the alkaline solution, the higher the compressive strength. Finally, if the sandstone was mixed with FA, the compressive strength further increased. In a following study, the same authors investigated the influence of the activator type and Pisha sandstone particle size [52]. Milled Pisha sandstone (mean diameter of 18.9 μm) yielded higher compressive strengths compared to un-milled stone particles (mean diameter of 111 μm). Moreover, the authors confirmed the strong influence of activator pH, obtaining higher mechanical properties using stronger alkaline activators: The best results were achieved by using NaOH (pH of 10.9) and Na_2_SiO_3_ (pH of 11.5) compared to other basic solutions whose pH was lower than 9.0.

Palmero et al. [46] obtained very good mechanical properties for alkali-activated granite pastes, which had flexural and compressive strengths of approximately 14 and 35 MPa, respectively, even without using any other ‘reactive’ components.

One-part geopolymers demonstrated their ability to develop high-mechanical-strength materials, too. In fact, Hassan et al. [54] reported good compressive strength for WBA/diatomite pastes. In particular, the highest compressive strength at 28 days (48 MPa) was measured for samples containing 21.7% of dry activator. Feng et al. [49] obtained a compressive strength of 44.2 MPa for one-part geopolymers prepared with thermally treated albite (at 1000 °C with 50% of NaOH), while a slightly lower compressive strength (i.e., 42.6 MPa) was measured for albite calcined with Na_2_CO_3_.

#### 2.2.3. Durability Properties of Hardened Materials

Only a few authors investigated the influence of alumino-silicate mineral additions on the durability of alkali-activated materials. In particular, two researches [47,48] investigated the role of alumino-silicates on crack formation, which is a well-known issue on both cementitious materials and geopolymers, whose effect can be effectively contrasted by the use of synthetic or natural fibers [83,84]. Only one work discusses the role of these minerals on the high-temperature stability [52].

Choi et al. [47] observed numerous cracks in alkali-activated GBFS samples due to slag’s high reactivity and a positive influence of granite addition on both crack formation and compressive strength. Tchadjié et al. [48] observed cracks in geopolymers prepared using fused granite waste and MK. The authors attributed crack formation to the release of free water, which did not participate in the geopolymerization process. Crack number and width decreased with increasing Na content in the fused granite waste, probably due to the formation of a soluble silicate phase that acted as a filler in the cracks. Moreover, the authors investigated the samples’ resistance to water immersion; by increasing the Na_2_O content in the fused granite waste, the presence of cracks after water immersion increased as well. However, the sample with the lowest Na_2_O content (i.e., 10 wt.%) completely dissolved, suggesting the need to further optimize the mixture’s composition.

**Figure 2:**
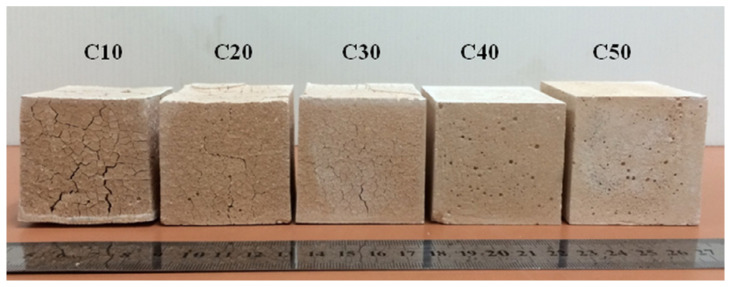
Physical appearance of metakaolin/cordierite geopolymers after firing at 800 °C/2 h, with increasing cordierite addition (from 0 to 50 wt.%). Reprinted from [53] under the license n. 4792970655838.

Hemra and Aungkavattana [53] tested the effect of high temperature exposure (800 °C for 2 h) on MK/cordierite samples. The authors found that with increasing cordierite addition, cracking phenomena were reduced (Figure 2), even for low cordierite amounts (i.e., 10 wt.%), while they were completely hindered at higher weight fractions (i.e., 40 and 50 wt.%). These last samples were also submitted to thermal shock resistance tests by placing the samples in a furnace pre-heated to 800 °C for 10 min and then immediately removed. The samples were able to support 15 cycles without cracking, even if their compressive strength was almost halved.

### 2.3. Role of Alumino-Silicate Minerals in the Alkali Activation Process

The role of alumino-silicate mineral waste on alkali-activated materials is still not completely clear. Some authors, in fact, attribute to these particles just a ‘filler’ effect. As an example, in Ref. [53], cordierite powder was added to MK (from 0 to 50 wt.%), showing a remarkable effect in improving thermal stability and thermal shock resistance, which was imputed to a filler effect of the particles, which reduced shrinkage and cracking.

However, most of the authors agreed on considering alumino-silicate minerals as not being inert in the alkali activation process and used several characterization techniques to demonstrate a possible role exerted by these mineral fines. As an example, Choi et al. [47] investigated the X-ray diffraction (XRD) phase composition of alkali-activated GBFS pastes containing granite sludge. The authors observed the formation of hydrotalcite (Mg_6_Al_2_CO_3_(OH)_16_·4(H_2_O)), as already observed in alkali-activated slag [85]. However, the formation of this phase was favored in the granite-containing samples, as compared to ‘pure’-GBFS materials, suggesting a contribution of granite powder in the alkali activation process. Clausi et al. [50,76] suggested a role of Pietra Serena fines used in the preparation of MK-based mortars. In fact, by microstructural Scanning Electron Microscopy (SEM) observations, larger quartz and feldspar grains (contained in Pietra Serena) evidenced an incipient dissolution, and a limited formation of an alumino-silicate gel from Pietra Serena sludge was demonstrated [76].

In order to improve the reactivity of alumino-silicate mineral fines under alkaline condition, thermal activation was tested. Feng et al. [49] applied a thermal activation to waste albite particles and heated albite/sodium hydroxide or sodium carbonate dry mixtures between 850 and 1150 °C. For temperatures higher than 1000 °C, an almost completely amorphous structure was identified by XRD analysis, providing in this way a highly reactive geopolymer precursor. Similarly, Tchadjié et al. [48] applied an alkali fusion process to waste granite/sodium hydroxide pellets at 550 °C. After this treatment, the mineral composition of granite was modified, as ascertained by XRD analysis. In fact, while the raw powder was composed of well-crystallized quartz, biotite, almandine, and albite phases, in the melt sample, a decrease of these peaks was observed in addition to the appearance of the sodium silicate phase. In addition, the XRD patterns of the fused materials showed the presence of a halo, attesting the formation of an amorphous phase and a consequent high reactivity under alkaline condition.

**Figure 3:**
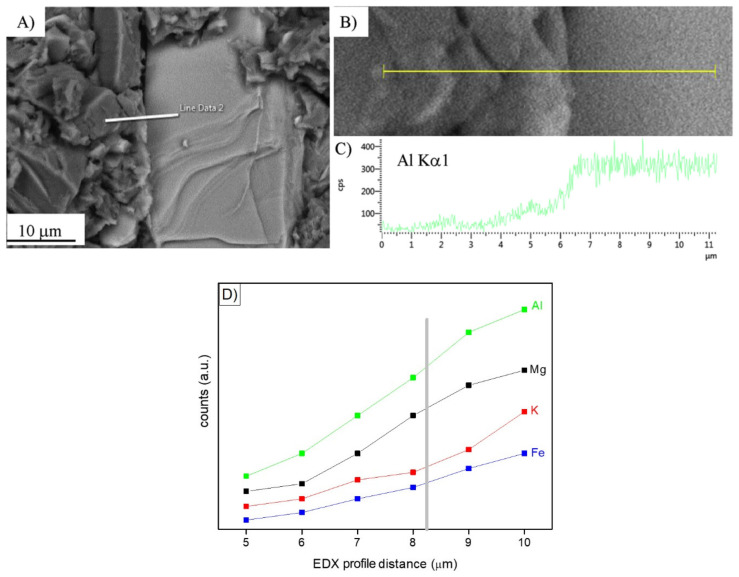
(**A**,**B**): Field emission scanning electron microscopy (FESEM) micrographs at different magnifications of a biotite grain in a one-year-aged sample; (**C**): Energy dispersive X-ray spectroscopy (EDX) profile related to Al Kα1 along the line shown in (**A**,**B**). Reprinted from [46] under the license n. 4792980067658. In (**D**), the Al, Mg, K, and Fe profile evolution at the grain–matrix interface is highlighted (unpublished image by the authors).

**Figure 4:**
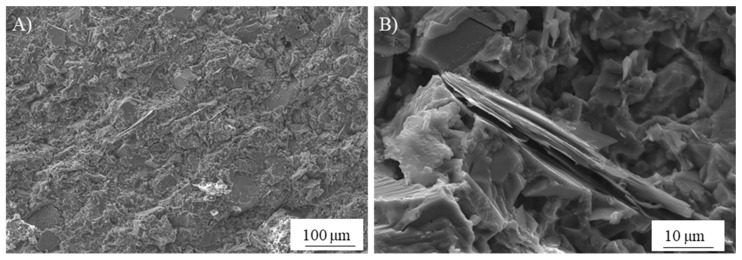
FESEM micrographs of the microstructure produced by the alkali activation of a pure alumina-silicate mud: (**A**) highly compact matrix and (**B**) detail of the undissolved particles (unpublished images by the authors).

In search of more environmentally friendly processes, some researchers tried to exploit alumino-silicate mineral fines as the only precursors of alkali-activated materials, without any thermal activation [46,51,52]. For instance, Palmero et al. [46] investigated the possibility of using granite mud as raw powder. The phase composition was investigated by XRD and phase quantification was carried out by Rietveld analysis for both raw powder and alkali-activated material. Compared to raw powder, a general decrease of the crystalline phases was observed; the biotite phase showed the highest weight decrease (44%), followed by albite (38%) and clinochlore (29.4%), while a lower reactivity was determined for microcline and quartz (around 22% for both phases). At the same time, an increase of the amorphous phase was determined. These results demonstrated a certain dissolution of crystalline alumino-silicate particles in a strong alkaline solution, and thus an active role in the alkali activation process. Further proof of the dissolution phenomena of alumina-silicate particles was obtained by field emission scanning electron microscopy equipped with energy dispersive X-ray spectroscopy (FESEM/EDX). An example is provided in Figure 3, showing an undissolved biotite grain surrounded by the matrix. Systematic EDX elemental profiles of the main elements were determined from the outer to the inner part of the grain, as shown by the white and yellow lines in Figure 3A,B). The Al Kα1 EDX profile is provided in Figure 3C, showing that the Al concentration was almost constant inside the grain, while it clearly decreased in correspondence with the biotite–matrix interface. However, though in lower concentration, all elements (Al, Fe, K, and Mg; see Figure 3D) were clearly detected in the matrix around the grain (a few microns from the interface), thus confirming the surface dissolution and the elemental diffusion in the surrounding binder. Through this mechanism, it was possible to produce materials characterized by a highly compact matrix (Figure 4A), with a good adhesion between the finer matrix and the undissolved particles (Figure 4B).

Li et al. [51,52] confirmed the possibility of alkali-activating a Chinese sandstone mainly composed of quartz and albite with minor amounts of calcite (CaO content was 8.02% in [51] and 5.10% in [52], respectively). However, in this case, the authors ascribed to calcite, and not to alumino-silicates, the main role in the geopolymer process; in fact, by means of XRD, Fourier-transform infrared spectroscopy (FT-IR), and thermo-gravimetric analysis coupled with derivative thermo-gravimetric analysis (TGA/DTG), the authors attested the formation of calcium silicate hydrates (C-S-H) in alkali-activated Pisha sandstone samples [51,52].

## 3. Alkali-Activated Materials Based on Carbonate Minerals

### 3.1. Role of Raw Materials, Activators, and Curing Conditions

The carbonate materials considered here are mainly composed of two minerals: Calcium carbonate (CaCO_3_) and calcium magnesium carbonate (CaMg(CO_3_)_2_). The first category includes marble (metamorphic rock), limestone, and travertine (sedimentary rocks), and is characterized by a CaO content between 35 and 55 wt.% (Table 3). The second group includes dolomite (sedimentary rock), in which MgO content varies approximately between 15 and 35 wt.%.

As stated in the introduction, most of the works considered the use of carbonate powders only as filler added to the raw powders traditionally used in the alkali activation process [50,55,56,57,58,59,60,61,62,63,64,65,66,67,68,69,70,71,72]; only two works investigated the use of carbonates alone [40,45]. In particular, carbonate fines have been used in combination with MK [50,55,57,67], FA [58,59,62,65,66,68], GBFS [56,59,61,65,68,69,70,72], and clays (bentonite [60], smectite [64], halloysite [71], undefined [65]).

The main difference among all the studied carbonate stone powders is the calcite and/or dolomite content, resulting in a different chemical composition that can be mainly expressed in terms of CaO and MgO content (Table 3). Indeed, articles can be summarized in three different groups according to the chemical nature of the starting minerals: mainly composed of calcite [45,55,56,57,58,60,63,64,65,66,67,68,69,70,71,72], with prevailing dolomite content [50,55,59,60,61,62] and a mixture of calcite and dolomite [72].

Concerning the calcite-rich group, Tekin [63] studied the possibility of using marble and travertine in combination with a waste volcanic tuff, a natural pozzolan containing zeolite. Marble’s and travertine’s chemical compositions were similar (Table 3), but travertine showed a higher porosity. Several formulations were investigated, in which marble or travertine ranged from ~20 to 80 wt.%, tuff being the remaining fraction [63]. Thakur et al. [66] prepared FA geopolymer bricks via extrusion, in which different marble fractions (i.e., from 10 to 80 wt.%) were added. Gao et al. [68] investigated ternary mixtures containing GBFS/FA/limestone with three different limestone contents: 10, 20, and 30 wt.%, respectively. Bayiha et al. [71] studied the alkali activation of thermally activated halloysite (a two-layered clay) with several limestone replacements (from 0% to 60%). Finally, Orteza-Zavala et al. [40] and Coppola et al. [45] used only calcite (limestone and marble, respectively) as raw powders in the alkali activation process.

In the case of dolomite-rich compositions, Cohen et al. [62] investigated the use of a quarry dust mainly composed of dolomite with calcite traces in combination with both cement and low-calcium FA. Several dolomite fractions (10, 20, 30, and 40 wt.%) as cement or FA replacements were investigated. Clausi et al. [50] investigated the possibility of using ornamental stone aggregates in the preparation of MK-based geopolymers for cultural heritage applications. The authors studied both a siliceous sandstone (Pietra Serena) and a dolostone (Pietra di Angera) with particles smaller than 0.5 mm, including fines.

Concerning mixed calcite–dolomite powders, Rakhimova et al. [72] investigated the role of three different limestone powders on the alkali activation of GBFS powders. In particular, the three powders were characterized by different calcite contents: 33% (with 66% dolomite and 1% quartz), 90% (with 9 wt.% quartz and 1 wt.% albite), and 100 wt.% [72]. Kürklü and Görhan [58] investigated the role of a quarry dust composed of calcite with dolomite traces as aggregate in geopolymers prepared from low-calcium FA (class F).

As regards the activating solution, Table 1 displays the general composition of the alkali activators used in the papers here reviewed, while Table 3 summarizes the main activating solution parameters (concentration, modulus, etc.) used in the case of carbonate-containing materials.

It can be easily observed that alkaline solutions containing sodium hydroxide (NaOH) and sodium silicate (Na_2_SiO_3_) are the most used [40,45,50,55,57,58,62,64,65,66,68,71]. Incidentally, it is worth noticing that, due to its reactivity properties as an alkaline activator, the use of sodium silicate is also well established in the cement and concrete industry, where it may act as a sealant, a densifying additive, or even a healing agent for self-healing applications [86,87,88,89]. Generally speaking, two parameters are mainly considered for the preparation of the activating solution: (i) The modulus M_s_ (i.e., the SiO_2_/Na_2_O molar ratio) for solutions containing both NaOH and Na_2_SiO_3_; (ii) the NaOH molar concentration, when only NaOH water solution is used [63].

Two studies [40,65] demonstrated the important effect of sodium silicate contained in the activating solution in increasing the materials performance. Salihoglu and Salihoglu [65] compared the behavior of marble sludge/fly ash geopolymers, activated using either a pure NaOH solution or a mixed NaOH/Na_2_SiO_3_ solution, and showed higher mechanical properties in the latter case. Similarly, Ortega-Zavala et al. [40] prepared alkaline solutions at three different SiO_2_/Na_2_O molar ratios: 0 (i.e., only NaOH, no sodium silicate), 1.0, and 1.5. Once again, the highest mechanical properties of alkali-activated limestone were achieved in the presence of sodium silicate.

**Figure 5:**
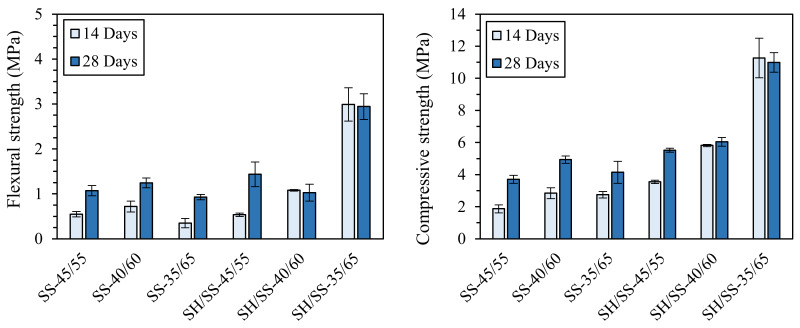
Flexural and compressive strengths of the different mixtures with different L/S ratios (i.e., 45/55, 40/60, and 35/65) prepared with and without NaOH (SH/SS and SS, respectively). Unpublished results by the authors.

Oppositely, in a previous work by Palmero and co-workers (unpublished results), the importance of NaOH in the activating solution was demonstrated. In fact, either a pure sodium silicate solution (SS, pH of 10.8) or a mixed NaOH/sodium silicate solution (SH/SS, pH of 12.7) was used for the activation of a carbonate mud. In this study, mixtures with different liquid-to-solid ratios (L/S) were investigated—precisely 45/55, 40/60, and 35/65—and cured for 14 or 28 days. The (unpublished) results achieved are displayed in Figure 5. It is possible to observe the key role played by NaOH in increasing the mechanical properties, since both flexural and compressive strengths of the NaOH-containing mixtures were higher than those of the specimens activated with only pure sodium silicate solution, suggesting a more effective species dissolution in a stronger alkaline medium. Furthermore, for NaOH-containing samples, the strengths increased by increasing the solid loading, while no meaningful differences were observed for the other mixtures. Finally, considering the influence of curing time, it is evident that the development of the mechanical properties for the mixtures without NaOH is delayed compared to the sodium-hydroxide-containing samples.

Similarly, Thakur et al. [66] prepared alkaline solutions based on NaOH and sodium metasilicate at two different NaOH molarities (2 and 4 M) for the activation of marble waste FA samples. In agreement with the results discussed above, materials prepared at the highest molar concentration (and therefore at the highest pH) showed higher mechanical properties and decreased water absorption.

Kürklü and Görhan [58] discussed the influence of both curing time (from 1 to 5 h, at 80 °C) and NaOH concentration (3, 6, and 12 M) on FA geopolymer mortars containing carbonate dust. In particular, the highest molar concentration was the most favorable condition in terms of apparent porosity and water absorption coefficient. At the same time, a longer curing time was more effective for the geopolymerization reactions to occur.

In [57], the combined effect of sodium and potassium hydroxides was investigated, as this study was based on the different roles played by these two alkaline metals in the geopolymerization process of calcined clays. Sodium, in fact, is known to increase the dissolution of amorphous phases, while potassium is known to promote a higher degree of polymerization reaction [90].

Interestingly, in some previous researches, sodium carbonate (Na_2_CO_3_) [59,69,70,72] was used to prepare the activating solution. Among activators, Na_2_CO_3_ is globally available and environmentally friendly. Moreover, Na_2_CO_3_ is easier to handle, characterized by lower pH, and cheaper than sodium silicate [91]. Yuan et al. [69,70] used sodium carbonate as an activator in GBFS/limestone geopolymers. Several GBFS replacements were investigated (5, 10, 15, and 30 wt.% in [69] and 10, 20, 30, 40, and 50 vol.% in [70]), and the obtained pastes were cured at room temperature and high RH (20 °C and >95%, respectively) [69,70]. Rakhimova et al. [72] exploited an alkaline solid waste as the activating ingredient; it was derived from the incineration of sewage of a petrochemical company and consisted mainly of Na_2_CO_3_ (91.4 wt.%) and NaOH (2.65 wt.%).

In [64], sodium citrate (Na_3_C_6_H_5_O_7_) was added to a sodium hydroxide–sodium metasilicate solution used to activate waste carbonate/calcined clay mixtures. The authors observed an improvement in the workability of the pastes due to citrate addition, leading to a less porous microstructure and higher mechanical properties of the hardened materials.

Some studies relate to the already mentioned one-part alkali-activated materials, in which the dry alkaline component is mixed with the raw powder so that only water is used to activate the mixture [56,60,61]. Abdel-Gawwad and Abo-El-Enein [56] mixed calcium carbonate and a sodium hydroxide solution (at different NaOH concentrations: 2%, 4%, and 6%, respectively) for the preparation of a dry activator to be used in combination with GBFS. Then, activation occurred by adding only water to the GBFS/dry activator mixtures. Peng et al. [60] investigated the possibility of preparing one-part alkali-activated materials using a mixture of bentonite, dolomite, and sodium carbonate. In particular, the authors prepared several mixtures of these three raw powders, which were then calcined at 1100 or 1200 °C. The calcination process resulted in the preparation of clinkers with several compounds (such as calcium aluminum oxide—Ca_3_Al_2_O_6_, belite—Ca_2_SiO_4_, and sodium iron oxide—NaFeO_2_ and MgO) that were hydrated by adding only water, as the alkali additive was already contained in the clinker. Yang et al. [30] prepared GBFS/calcined dolomite blends (2, 6, and 10 wt.% of the GBFS) for the preparation of one-part sodium carbonate activated pastes at different Na_2_CO_3_ amounts (10 and 15 wt.%).

A further key parameter in property development is curing, as it affects alkali activation reactions and material microstructure. A summary of the curing conditions used in carbonate-containing alkali-activated materials is given in Table 3.

As a common approach followed by several authors, fresh pastes are submitted to a short curing period (typically between 1 and 24 h) in an oven at temperatures between approximately 40 and 80 °C [40,45,55,56,57,58,60,62,63,66,67]. In some other works, samples were exposed to room temperature during the whole curing period [50,59,61,63,64,65,68,69,70,72]. The curing atmosphere was a key parameter, too, and several authors cured samples under wet conditions (90%–100% relative humidity) [40,45,50,56,59,61,62,63,64,68,69,70,72] or directly immersed under water [45]. When samples are cured in high-humidity conditions, or even water-cured, two opposite behaviors have been described in literature: on one side, this curing condition promotes the formation of C-S-H species, thus enhancing the mechanical properties and durability [45,63,64]; on the other side, it slows down the geopolymerization process and induces the formation of cracks in the cured samples [45,63,64]. As an example, Valentini et al. [64] cured clay/marble samples for 24 h at 95% RH, followed by 20 days of dry-curing or water immersion. The authors observed that if the same samples were cured under water, several macrocracks appeared due to volume expansion. Tekin [63] investigated several curing conditions: laboratory (22 °C and 40% RH) and heat-curing (45 or 75 °C for 24 h). Then, in all three cases, half of the specimens were cured in wet conditions (>95% RH) and the other half kept in laboratory conditions (35% RH) up to the specimens’ testing. Heat-curing at 75 °C increased the early strength of the pastes, but resulted in crack formation. Therefore, the optimal curing temperature suggested was 45 °C. In addition, the author observed that wet curing was not favorable for the geopolymerization reactions, and therefore for the development of the mechanical properties. Coppola et al. [45] investigated the role of curing conditions on alkali-activated marble sludge. In particular, after a short curing time at 60 °C under wet atmosphere, different curing environments were tested: air-curing (RH = 18% ± 2%), humid-curing (RH = 95% ± 2%), and water-immersion, all of them at 20 °C. The most favorable condition for C-S-H development and mechanical property increase was air-curing, suggesting an important role played by both short-time wet-curing (probably for hydrated species formation) and longer-time air-curing (for gel formation and polymerization).

### 3.2. Properties of Fresh and Hardened Materials

#### 3.2.1. Fresh Mixture Properties

The mix composition and proportions play a key role on workability and casting behavior, which, in turn, affect the microstructure of the final product and its macroscopic mechanical properties. However, the carbonate addition appears to have controversial effects on the properties of fresh mixtures, as observed by different researchers.

On one side, Rakhimova et al. [72] observed that the consistency of alkali-activated pastes was not affected by GBFS replacement with limestone particles of different fineness. Gao et al. [68] observed a negligible effect of limestone on the initial and final setting time of slag–fly ash–limestone mixtures. Similar results were achieved by Aboulayt et al. [57], suggesting that the presence of calcium carbonate had no influence on MK alkali activation and acted as an inactive filler due its poor solubility in alkaline medium.

On the other side, other researches underlined that the addition of carbonate fines could affect the fresh mixture properties, even if different trends are described.

Tekin [63] investigated the setting time of alkali-activated tuff pastes added with marble or travertine powders. Both initial and final setting times increased with increasing travertine or marble fraction, even if these values decreased with increasing NaOH concentration. Accordingly, Bayiha et al. [71] reported a significant increase of setting time for MK-based geopolymers with increasing limestone contents. Such setting time delay was imputed to the reduced amount of reactive MK in the formulations, but also to the enhanced fluidity of the paste and a consequent reduction of the contacts between MK particles. Yuan et al. [69,70] reported an improved flowability at increasing GBFS replacements with limestone, thanks to the lower water demand of the latter compared to GBFS. A similar behavior was reported by Gao et al. [68], describing an increased slump flow with increasing limestone content in alkali-activated slag–fly ash–limestone mixtures. The authors imputed this behavior to a higher flowability of limestone into alkaline solutions as compared to the other constituents, and also suggested a better particle packing of limestone-containing pastes, meaning more available water to lubricate the particles.

Contrary to such previous works, other studies report a negative role played by carbonate fines on the fresh properties. For instance, Kürklü and Görhan [58] investigated the rheological properties of FA-based geopolymer mortars prepared using a calcite quarry dust as fine aggregate. The authors observed a negative effect of the dust on the paste workability due to calcite of a very fine size. The mixtures were thus optimized by replacing a part of the dust with silica sand with larger particle size. Cohen et al. [62] used a mini-flow table test to study the influence of dolomite fines on the fresh properties of two binders: cement and fly ash. The authors varied water (in cement/dolomite mixtures) or activator (in cement/fly ash mixtures) content in order to keep constant the water/binder or activator/binder ratio. Mixture flow decreased with increasing dolomite content due to the reduced water/dry solid ratio. A further negative effect on slurry fluidity was imputed to the irregular shape of the dolomite particles. This negative effect was more pronounced with dolomite substitution higher than 20 wt.%, severely compromising the good flowability induced by the round-shaped fly ash particles.

Concerning one-part geopolymers, Yang et al. [61] measured a rapid decrease of the setting time (both initial and final) using the Vicat needle penetration tests with increasing calcined dolomite content in sodium carbonate activated GBFS pastes, regardless of Na_2_CO_3_ content. In GBFS-based geopolymers developed by Abdel-Gawwad and Abo-El-Enein [56] (in which the activator was composed of calcium carbonate and a sodium hydroxide mixtures), a decrease of initial and final setting time with increasing sodium hydroxide contents was observed. This behavior was imputed to the formation of more calcium hydroxide, acting as an accelerator of the hydration reactions. Calcium carbonate was identified as a nucleation site, promoting the formation of hydrated products and thus shortening the setting times.

#### 3.2.2. Mechanical Properties of Hardened Materials

A literature survey allowed us to distinguish three main behaviors concerning the mechanical properties of alkali-activated materials containing carbonate minerals.

First, an increase of mechanical properties by increasing the carbonate mineral addition has been described in [56,61,62,64,65,68,71].

For instance, Salihoglu and Salihoglu [65] reported an increase of compressive strength of FA-based geopolymers: Indeed, compressive strength increased from 16 and 30 MPa, moving from pure FA to 50 wt.% FA–marble mixtures. To explain this increase, the authors suggested a role of CaO contained in the marble in accelerating the hardening of geopolymer samples thanks to C-S-H gel formation and the consequent increase of the mechanical strength. Valentini et al. [64] obtained a considerable increase of compressive strength (+110%, 60.7 MPa) when 25 wt.% calcined smectite clay was replaced with the same amount of waste marble powder and activated with a solution composed of sodium hydroxide, sodium metasilicate (Na_2_SiO_3_·5H_2_O), and sodium citrate (Na_3_C_6_H_5_O_7_). However, in these papers, a single mixture composition was investigated, and, therefore, a trend of mechanical properties versus marble content was not established. Gao et al. [68] reported a slight but continuous compressive strength increase for GBFS/FA/limestone blends at increasing limestone contents (up to 30 wt.%). This behavior was observed in samples cured for both 7 and 28 days, and was imputed to the filler effect of limestone powder. Limestone fine particles, in fact, reduced the total porosity by acting as micro-aggregates, and thus increased the strength. Cohen et al. [62] obtained a considerable increase of compressive strength of fly ash/dolomite mixtures at increasing dolomite contents. In particular, a compressive strength of approximately 40 MPa was obtained at 30 wt.% dolomite and almost the same value for further dolomite addition (40 wt.%), suggesting that a threshold was reached. The authors attributed this strength increase to a mechanical anchoring between the matrix and dolomite particles thanks to the irregular shape of the latter. Moreover, a dense interfacial transition zone was observed, indicating a good compatibility between the particles and the matrix. Yang et al. [61] produced one-part alkali-activated GBFS/calcined dolomite samples, and observed a progressive increase of the compressive strength by increasing the calcined dolomite amount (from 2 to 10 wt.%). In particular, the highest obtained compressive strength was ~42 MPa for 10 wt.% calcined dolomite activated with 10 wt.% Na_2_CO_3_.

The second most common behavior observed in the works analyzed here relates to the achievement of an optimum in mechanical properties depending on the amount of carbonate fines [55,57,67,69,70,72].

For example, Aboulayt et al. [57] replaced MK with increasing percentages of calcite (2, 4, 6, 8, 10, and 12 wt.%, respectively): All mixtures—with the only exception of the 12 wt.% substituted MK—showed a higher compressive strength as compared to neat MK, with the maximum strength (approximately +15%) at 6 wt.% calcite. A slight increase of flexural strength at increasing calcite replacements was observed as well, but, in this case, a clear trend could not be stated. Similarly, Yip et al. [55] observed a certain improvement of compressive strength by adding 20 wt.% calcite to MK, followed by a sharp decrease for further additions (from 40% to 100%). The authors compared the mechanical behavior of MK/calcite and MK/dolomite pastes under the same alkaline activation and curing conditions. The calcite contribution to the mechanical strength was higher than the dolomite one, despite the fact that this latter mineral is characterized by higher hardness. Thus, a ‘simple’ filler role played by these mineral powders was excluded, and the lower mechanical performance achieved by the MK/dolomite system was attributed to either a lower dissolution degree of calcium or to different surface properties of calcite and dolomite [92,93], which may affect the binding of the minerals to the geopolymer gel. Rakhimova et al. [72] investigated the influence of limestone composition, content, and size in blends with GBFS. Three types of limestone (differing in mineralogical composition, see Section 3.1 and Table 3) were milled to achieve specific surface areas of 200, 400, and 600 m^2^/kg, respectively, corresponding to increasing particle fineness. Compressive strength increased by decreasing particle size, and maximum strength at 30–40 wt.% limestone was achieved for all three limestone types. Finally, the highest strength was achieved for pure-calcite limestone, confirming a stronger effect of this phase compared to dolomite and quartz. The effect of calcite on MK-based geopolymer properties was confirmed by Cwirzen et al. [67]. Here, MK was replaced by various limestone fractions (from 30 to 70 wt.%), and a certain increase of compressive strength was achieved at 30 and 50 wt.% limestone replacements (specific optimal values were functions of curing time, curing temperature, and NaOH concentration). Similarly, Bayiha et al. [71] obtained a slight increase of seven-day compressive strength for MK/limestone geopolymers with increasing limestone content of up to 15 wt.%, followed by a decrease for further additions (up to 60 wt.%). Moreover, geopolymer pastes containing limestone developed their mechanical properties more rapidly, as is evident when comparing 7 and 28 day compressive strengths [71]. Thakur et al. [66] observed an increase of compressive strength for FA/marble waste geopolymers of up to 60 wt.% of marble, whereas beyond this value, i.e., at 70 and 80 wt.%, compressive strength decreased due to the lack of silica and alumina (deriving from FA) necessary for the geopolymer network formation. Yuan et al. [69,70] investigated the alkali activation of GBFS/limestone mixtures with limestone ranging from 5 to 30 wt.%, and, by using Na_2_CO_3_ as an activator, observed optimal strength at 10 wt.% limestone. Limestone particles accelerated and intensified the reactions, acting as nucleation sites, but with the highest limestone contents, a dilution effect (due to GBFS replacement by limestone) occurred, and the strength decreased again [70]. Abdel-Gawwad and Abo-El-Enein [56] investigated the compressive strength of one-part GBFS-based geopolymers blended with a dry activator composed of a dried mixture of sodium hydroxide and calcium carbonate. The strength progressively increased moving from 0 to 10 wt.% of calcium carbonate in the activator (achieving the maximum strength of approximately 70 MPa at 28 days of curing) and decreased again at 15 wt.% calcite. The authors explained the behavior by suggesting a role of the produced calcium hydroxide in accelerating the geopolymerization reactions and in producing hydration products (C-S-H and calcium aluminate hydrates, C-A-H) via pozzolanic reactions.

As a third behavior, a few reports describe a negative role of carbonate fines on the mechanical properties of alkali-activated materials [50,63,70].

For instance, Yuan et al. [70] observed a slight but progressive decrease of compressive strength by increasing the limestone content (from 10 to 50 vol.%) in GBFBS/limestone mixtures. This trend was observed for samples cured for both 7 and 28 days, while at 91 days of curing, the optimal strength was recorded at 10 wt.% limestone. Clausi et al. [50] compared MK-based geopolymers containing different aggregates: Standard siliceous sand, Pietra Serena (sandstone), and Pietra di Angera (dolostone) sands. The authors reported a sharp decrease of mechanical properties for the formulations containing the two ornamental stones. In the case of Pietra di Angera, larger pores were found in the interfacial zone between the aggregates and the geopolymer gel. Moreover, mortars containing Pietra di Angera aggregates presented a network of micro-cracks in the geopolymeric matrix and along the matrix–aggregate interfaces [50]. Kürklü and Görhan [58] used a carbonate quarry dust as aggregate in the preparation of FA-based geopolymer mortars, and compared these materials with samples in which one third of the dust was replaced by standard quartz sand. Under the optimal conditions, i.e., 12 M NaOH and 5 h curing, a slight decrease of flexural and compressive strength was observed for the pure-quarry dust aggregate.

Finally, in two very recent works, good mechanical results were also obtained in the case of alkali activation of ‘pure’ carbonate fines used without any further addition [40,45]. Ortega-Zavala et al. [40] obtained pastes with compressive strengths at 28 days ranging between 10 and 15 MPa, depending on the Na_2_O content. Moreover, a further slight increase of mechanical properties was found for longer curing periods [40]. Similarly, Coppola et al. [45] studied the alkali activation of waste marble sludge, reaching a compressive strength of approximately 12 and 40 MPa for moisture-cured and air-cured samples, respectively.

#### 3.2.3. Durability Properties of Hardened Materials

The durability of alkali-activated materials containing carbonate fines was investigated by water immersion and absorption tests [45,56,64,66,71] by observing efflorescence occurrence [45,50,63,71], shrinkage [45,55,70,72], and microstructure [45,70].

**Figure 6:**
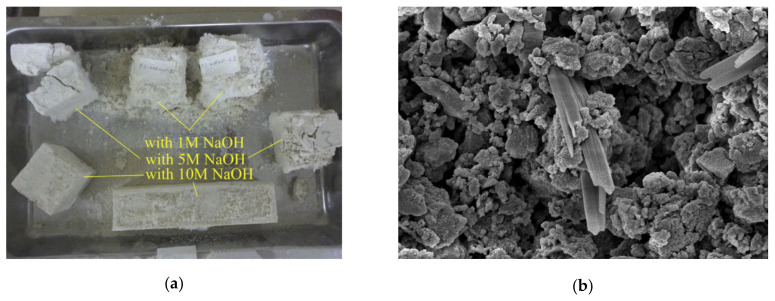
(**a**) Tuff/marble–travertine samples produced at different NaOH molar concentrations and submitted to water absorption tests. Reprinted from [63] under the license n. 4792980356522; (**b**) FESEM micrograph of hydrated sodium carbonate efflorescence (same materials used in [45] and humid-curing).

The behavior in the presence of water is extremely important for construction materials. Considering water absorption, different authors claimed a positive effect played by sodium and calcium carbonate [56], marble [66], or limestone [71] due to their filler effect. For instance, Bayiha et al. [71] reported a decrease of absorbed water by increasing limestone content (up to 45 wt.%) in MK geopolymers, while a further increase (60 wt.%) was detrimental. In the first case, limestone contributed to paste densification by reducing capillary pores. On the contrary, at higher limestone contents, MK was not sufficient to provide the polycondensation reactions, and unreacted water evaporation resulted in formation of pores [71]. In FA/marble blends, Thakur et al. [66] observed that the higher the marble content, the lower the water absorption, thanks to the lower porosity of the samples. Similarly, Tekin [63] observed a lower apparent porosity and a consequent higher resistance to water absorption in waste marble and waste travertine-added pozzolan compared to the pure material. The author found the optimal conditions by also properly setting the NaOH molar concentration in the alkaline solution; as depicted in Figure 6a, only a strong alkaline medium (NaOH 10 M) allowed the full contrast of the water-induced degradation and failure.

A well-known issue of geopolymers and alkali-activated materials is the efflorescence occurrence under wet conditions, which—in some cases—can lead to samples’ disintegration. The presence of carbonates seems to play a positive effect, limiting efflorescence appearance [50,71]. Clausi et al. [50] observed the positive effects of both Pietra di Angera and Pietra Serena in inhibiting the efflorescence occurrence in MK-based geopolymers. The authors affirmed that the presence of aggregates rich in aluminum (Pietra Serena) and calcium (Pietra di Angera) increased the crosslinking degree in the geopolymer gel, reducing Na^+^ cations’ mobility and, thus, sodium carbonate formation (i.e., efflorescence) [50]. Anyway, curing conditions play an important role in efflorescence occurrence; for instance, Coppola et al. [45] observed efflorescence formation in wet-cured carbonate geopolymers (Figure 6b), while in air-cured and water immersed samples, it did not appear.

As regards dimensional stability, a different effect played by the carbonate addition was reported. In fact, a reduction of shrinkage and cracking phenomena due to the presence of limestone in GFBS activated materials was observed by Rakhimova et al. [72].

On the other hand, most of the works reviewed here reported an increase of drying shrinkage with increasing mineral addition [55,70,71], which was mainly imputed to the presence of unreacted water. Yip et al. [55] evaluated the relative shrinkage of alkali-activated MK/calcite blends, obtaining an approximatively linear increase of shrinkage with increasing calcite content. For high-calcite samples (MK substitution higher than 60%), the authors observed the presence of some voids, imputed to the evaporation of unreacted water. A similar behavior was observed by Bayiha et al. [71] in MK/limestone geopolymers, who found an increase of shrinkage with increasing limestone addition. Once again, the reduced geopolymerization degree of the mixtures compared to neat MK lead to higher free water and, consequently, to larger shrinkage. Moreover, the shrinkage decreased with increasing NaOH molar concentration (from 5 to 8 M), strengthening the hypothesis that the shrinkage is strongly correlated with material reactivity.

Yuan et al. [70] investigated both autogenous and drying shrinkage of GBFS/limestone alkali-activated pastes. Autogenous shrinkage—which is correlated with the occurrence of chemical reactions during the first curing days—increased at low limestone additions (up to 30 vol.%). The authors correlated this behavior with the heat released, suggesting intensified reactions towards the formation of calcium aluminum silicate hydrate C-A-S-H gel due to the presence of moderate limestone amounts. The increase of drying shrinkage at increasing limestone contents was again correlated with more free water due to unreacted products, and with the dehydration from some crystalline phases (such as gaylussite and natron) produced during the alkali activation process.

**Figure 7:**
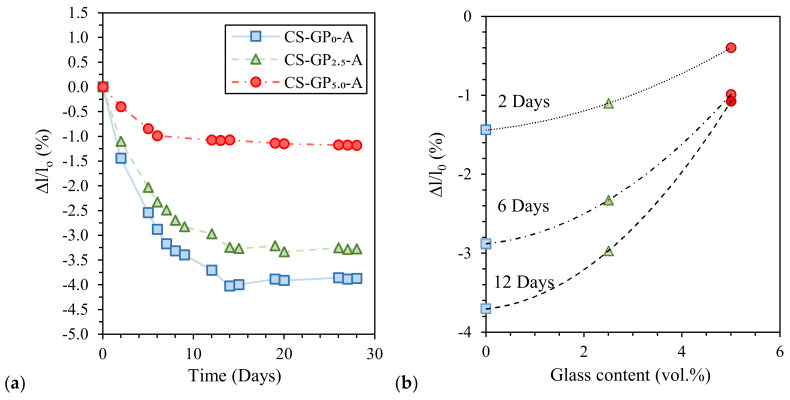
Length variation of air-cured alkali-activated marble sludge specimens: (**a**) Influence of glass addition (GP_0_ = no glass, GP_2.5_ = 2.5 vol.% of glass, and GP_5.0_ = 5.0 vol.% of glass) and (**b**) shrinkage at different curing times. Readapted from [45] under the license number 4793000364236.

Finally, Coppola et al. [45] investigated the influence of curing conditions and waste glass addition on the linear shrinkage of alkali-activated marble sludge. Air-cured pastes exhibited the highest shrinkage compared to the humid- and water-cured ones (Figure 7a) due to samples’ drying during curing at low RH (18% ± 2%). Moreover, waste glass addition significantly reduces paste shrinkage (proportionally to glass content) and anticipates length stationarity (Figure 7b). Interestingly, waste glass addition also influences water resistance after immersion, avoiding sample cracking, and mechanical properties, thanks to the provision of further silica [45].

Finally, discussing durability, the poor acid resistance of carbonate-rich geopolymers has to be mentioned. In this frame, Cohen et al. [62] investigated the chemical resistance of alkali-activated dolomite/cement and dolomite/fly ash samples through their immersion in 5% sulfuric acid solution for 100 days. The authors observed a rapid disintegration of the samples containing dolomite due to the rapid dissolution of this mineral in acid solutions. The rate of disintegration of these samples was much higher than those of pure FA or dolomite/cement mixtures.

### 3.3. Role of Carbonate Fines in the Alkali Activation Process

The role of carbonates in the alkali-activation process is still not clear and highly debated in literature.

Some authors suggested that carbonate minerals provided only a filler effect [50,57,62,72], while other works recognized an active role of calcium carbonate in producing some of the final reaction products [43,61,66,67,76]. Finally, in some cases, authors declared a combination of these two effects [52,55,64,65].

Concerning the physical effects exerted by carbonate particles, such as filler or particle reinforcement, Cohen et al. [62] carried out several microstructural observations of FA/dolomite mixtures and suggested a role of dolomite particles in mechanical anchoring to the matrix due to their unsymmetrical shape and complex morphology. Aboulayt et al. [57] claimed that calcite acted as an inactive filler in replacement of MK. Rakhimova et al. [72] supported this theory, but suggested the role of a “physically active” mineral; in fact, even if not taking part in the reactions, limestone affected the final properties by acting as a nucleation site and accelerating the reactions [72]. This theory was confirmed by other authors, as reported in the following. Clausi et al. [50] affirmed that no meaningful influence was exerted by dolostone aggregates in MK-based geopolymers, even if small amounts of Ca and Mg were incorporated in the geopolymer matrix. However, Ca and Mg concentrations were not sufficiently high to form C-S-H and other reaction products.

On the other hand, in the frame of an ‘active’ role played by calcium-based minerals, Thakur et al. [66] used a silica-rich marble powder for the preparation of FA-based geopolymers. The authors attributed a role to both silica and calcium contained in the waste particles; dissolution of the silica resulted in the formation of strong interfacial bonding between matrix and particles, while calcium favored alumino-silicate dissolution by locally raising the pH and, therefore, geopolymer reactions. The authors observed a significant increase of compressive strength with increasing marble content due to the formation of covalent bonds between marble particles and the FA matrix, with a positive effect on the distribution of the applied load onto the geopolymer matrix. Yang et al. [61] investigated the influence of calcined dolomite on the one-part sodium carbonate activated GBFS pastes. Calcined dolomite provided Ca(OH)_2_, CaO, and MgO that reacted and promoted GBFS activation; indeed, a higher extent of alkali activation was found in pastes with high calcined dolomite contents. In particular, Mg^2+^ reacted with Al^3+^ ions, consuming $CO_{3}^{2-}$ and forming hydrotalcite, Mg_6_Al_2_CO_3_(OH)_16_·4H_2_O. Hydrotalcite formation allowed the release of OH- ions that increased the solution’s pH and promoted further GBFS dissolution, resulting in the formation of more C-A-S-H gel phase. Finally, the higher the calcined dolomite content, the lower the average pore diameter, indicating a pore refinement effect [61].

**Figure 8:**
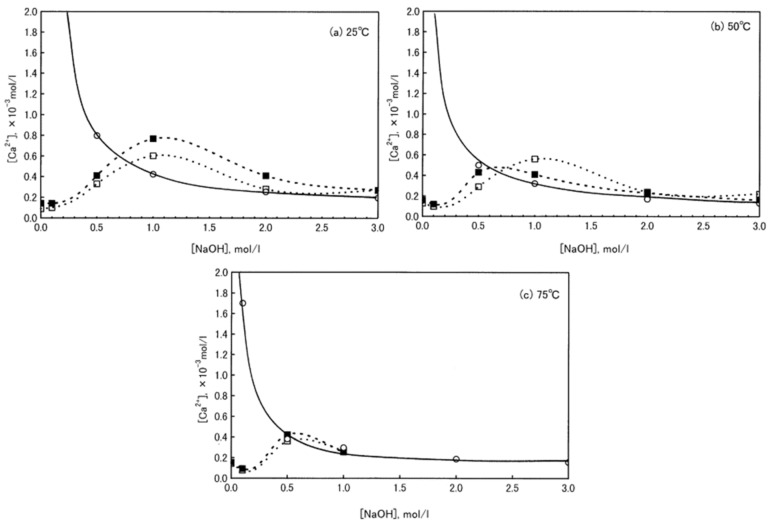
Solubility of Ca(OH)_2_, calcite, and aragonite in NaOH solution at 25 (**a**), 50 (**b**), and 75 °C (**c**); ○: Ca(OH)_2_, □: Calcite, ■: Aragonite. Readapted from [94] under the license n. 4793000615585.

For a better understanding of the role played by carbonates on the alkali activation process, first, the dissolution behavior of calcium carbonate particles under strong alkaline conditions has to be clarified. Ca^2+^ leaching from calcite and aragonite particles at different NaOH molar concentrations was investigated by Konno et al. [94] (Figure 8). It can be observed that Ca^2+^ release strongly depends on NaOH molar concentration by reaching a maximum at 1 M NaOH, at 25 and 50 °C, whereas, at higher temperatures (75 °C), this maximum is reached at 0.5 M NaOH. The authors explained this behavior by considering that calcite (and aragonite) are easily converted into Ca(OH)_2_ for NaOH concentrations higher than 2 M. Therefore, this study confirms not only calcite dissolution in alkaline medium, but also calcium hydroxide formation.

**Figure 9:**
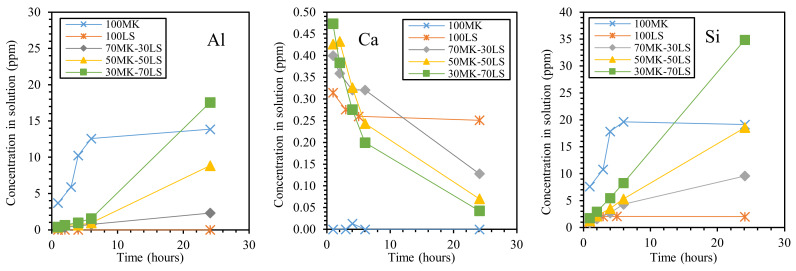
Effect of dissolution time and limestone (LS)/metakaolin (MK) ratio on leaching of each element from metakaolin–limestone blends. Redrawn from [67] under the license n. 4793200068229.

An important contribution to the understanding of the alumino-silicate/carbonate interactions under strong alkaline conditions was provided by Cwirzen et al. [67], who demonstrated by means of leaching tests that the presence of limestone (LS) influenced Si and Al dissolution from MK. Pure limestone and metakaolin as well as three limestone/metakaolin mixtures were investigated (LS/MK 30/70, 50/50, and 70/30), and the leaching of Al, Si, and Ca ions was determined as a function of leaching time (Figure 9). It can be observed that both Al and Si (leached from metakaolin) showed their highest values after 24 h of leaching, at the highest limestone concentration (LS/MK 70/30); these values are even higher than for pure MK. This behavior was explained by suggesting a chemical interaction exerted by limestone, which enhances the solubility of Al and Si. As a second explanation, the authors suggested a higher degree of delamination of the metakaolin particles in the mixtures, resulting in a higher effective alkali/metakaolin ratio. The solubility of Ca ions was low, in agreement with [94], and decreased with time, probably due to solution saturation effects. In addition, it seems that a small amount of Ca released from LS at an early age hindered the initial release of Al and Si from MK, even if the authors believe that more complex mechanisms are responsible for this behavior.

Furthermore, some recent studies explored the role of Al in C-S-H gel formation [95,96,97,98,99,100]. In a particular way, Kapeluszna et al. [95] investigated the influence of Al introduction into C-S-H gel in the presence of sodium hydroxide (pH 13), and demonstrated an increase of the amorphousness and of the bound water in C-A-S-H gel compared to the reference C-S-H. The authors demonstrated the key role played by the Ca/Si ratio on the Al behavior, suggesting that low-calcium systems promote incorporation of Al into C-A-S-H structures.

As stated above, several researches claim a combined effect of carbonates, meaning a twofold role as filler and ‘active’ component in the alkali activation process. In this frame, Yip et al. [55] observed that calcite was not chemically inert, and suggested different roles played by calcium in MK-based geopolymers: (1) reaction with silica to form amorphous C-S-H; (2) precipitation under an alkaline environment, forming calcium hydroxide, Ca(OH)_2_, which could transform into CaCO_3_ by carbonation due to atmospheric CO_2_; (3) reinforcement of the matrix, acting as a surface-bound physical filler, when added up to 20%; (4) micro-aggregates at a higher substitution degree. The authors concluded that the latter hypothesis was the most likely, and recognized a negative role played by the unreacted calcite particles on strength development, as they hindered the gel network continuity [55]. Salihoglu and Salihoglu [65] attributed the increase of compressive strength observed in marble/FA and marble/slag mixtures to the combined effect of pore refinement (i.e., filler effect) and C-S-H formation. Similarly, in [64], a double effect of waste calcium carbonate in calcined clay was postulated. In fact, according to the authors, the formation of C-A-S-H products besides the expected sodium aluminum silicate hydrate (N-A-S-H) ones [64] was confirmed by means of SEM-EDX analysis. Moreover, a micro-filler effect exerted by the unreacted calcined particles, contributing to mechanical strength development, was declared. In agreement with these two works, Bayiha et al. [71] suggested the formation of C-S-H in MK/limestone mixtures, providing a contextual increase of compressive strength of up to 45 wt.% limestone content. FT-IR analysis confirmed a positive effect due to limestone addition on geopolymer gel formation because the signal corresponding to the gel (between 950 and 1000 cm^−1^) increased with increasing limestone content. The authors concluded that partial dissolution of finely ground limestone occurred, leading to the formation of calcium silicate hydrates that are able to fill the geopolymer binder pores. At the same time, unreacted limestone particles acted as fillers, enhancing the densification of the pastes and reducing water absorption. Abdel-Gawwad and Abo-El-Enein [56] ascribed a role to calcium hydroxide (formed by the reaction of sodium hydroxide and calcium carbonate, see Equation (1) [94]) in increasing the leaching of Ca, Si, and Al ions from GBFS, and, consequently, in increasing the geopolymerization rate. The authors attributed to the undissolved calcium carbonate grains the role of nucleation sites, but, at the same time, they also regarded them as fillers able to reduce the porosity and increase the mechanical properties.

(1) $$2NaOH\left(aq.\right)+CaCO_{3}⇆Na_{2}CO_{3}+Ca{\left(OH\right)}_{2}$$

Once formed, calcium hydroxide can react via pozzolanic reactions [101] to form hydration products (C-S-H and/or C-A-H and C-S-A-H), according to Equation (2) (Ca/Si ratio and water molecules can be different):

Ca(OH)_2_ + H_4_SiO_4_ (or Si(OH)_4_) → CaH_2_SiO_4_·2H_2_O (2)

The possibility of pozzolanic reactions was also confirmed by Tekin [63], where a natural pozzolan was blended with both marble and travertine.

**Figure 10:**
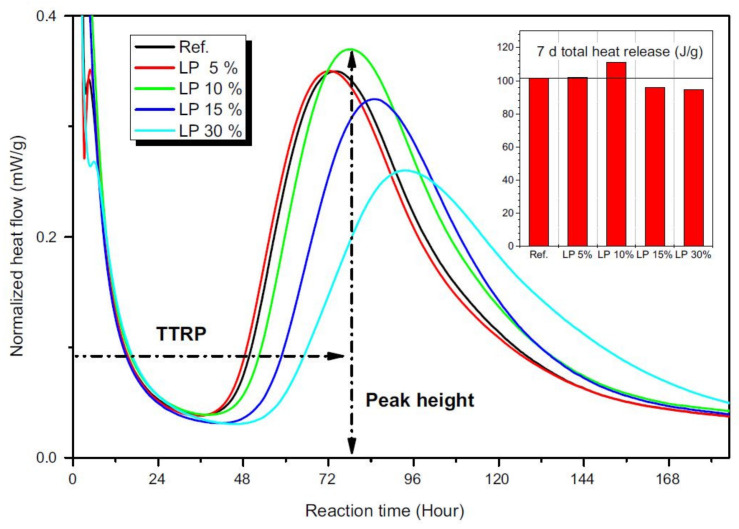
Heat flow normalized by mass of granulated blast furnace slag (GBFS) [69].

Finally, some authors demonstrated that the presence of calcium carbonate minerals influences the alkali activation reactions not only in terms of reaction products, but also in terms of dissolution kinetics. Gao et al. [68] investigated the behavior of GBFS/FA/limestone ternary blends and confirmed the role of limestone particles as nucleation sites, as already suggested in [56], for the formation and growth or reaction products. In fact, by means of isothermal calorimetric studies, reaction kinetics were investigated. By increasing limestone content, the heat flow peak progressively shifted towards shorter times with higher intensities, confirming that the alkali activation reactions of GBFS/FA mixtures were accelerated by limestone. Moreover, the authors confirmed the filler effect exerted by limestone particles, which were able to reduce the total porosity working as micro-aggregates. These two phenomena and the contextual possibility that some Ca^2+^ ions are released from limestone and participate in the reactions are responsible for the higher mechanical properties achieved in limestone-containing mixtures. A similar study was carried out by Yuan et al. [69], still by means of isothermal calorimetry, leading to the determination of the optimal limestone content in GFBS/limestone mixtures. As shown in Figure 10, low limestone additions (5–10 wt.%) had almost no influence on the heat peak position, meaning that the same C-S-H gel structure was formed regardless the limestone addition. However, at 10 wt.% limestone, an increase of the total heat release peak was observed, indicating an increase of the reaction products. On the other hand, further limestone additions of up to 30 wt.% decreased and delayed the formation of hydration products. In fact, an excess amount of limestone inhibited further Ca ions’ release from the slag.

As regards the role of limestone, some authors support both the hypothesis of nucleation site, as confirmed by calorimetric analysis, and a chemical effect on the reaction process [69]. Indeed, samples cured for 7 and 28 days showed the presence of gaylussite (NaCa(CO_3_)_2_·5H_2_O) and hydrotalcite (Mg_6_Al_2_CO_3_(OH)_16_·4(H_2_O)), in addition to calcite. The formation of these phases supports a chemical reaction among limestone particles and a sodium-carbonate-based activator. With longer curing stages, gaylussite converted into natron (Na_2_CO_3_.10H_2_O) and provided further hydrotalcite [69,70]. At later stages (28–180 days) natron is transformed into calcite, pirssonite (Na_2_CO_3_.5H_2_O), or thermonatrite (Na_2_CO_3_.H_2_O), releasing free water. The authors suggested that limestone powder chemically affects the decrystallization of gaylussite and that natron formation and transformation increases the mechanical properties [69]. Due to the consumption of Ca^2+^ and $CO_{3}^{2-}$ ions in the previous reactions, the hydrolysis of CaCO_3_ continued during long curing times, providing further Ca^2+^ for C-A-S-H gel formation [69]. Dolgaleva et al. [102] proposed the following reaction in the basic region (6.7 < pH < 13):

(3) $$CaCO_{3}+OH^{-}⇆CaOH^{+}+CO_{3}^{2-}$$

In the two studies regarding the alkali activation of carbonates as the main active ingredient, the authors confirmed the participation of Ca^2+^ ions in the formation of C-S-H. In particular, Ortega-Zavala et al. [40] demonstrated via FT-IR and ^29^Si magic angle spinning nuclear magnetic resonance (^29^Si MAS-NMR) analysis the presence of SiO_4_ tetrahedra in different coordination statuses, mainly Q2 and Q3, depending on the Ms. Similarly, Coppola et al. [45] obtained higher mechanical properties for air-cured samples thanks to the higher degree of polymerization obtained in this curing regimen. The authors found a more intense band in the FT-IR spectra attributed to C-S-H (around 950–970 cm^−1^), shifted toward higher wavenumbers compared to the samples cured in the other investigated curing environments [45]. Moreover, a further confirmation of a more condensed gel network was also derived from X-ray photoelectron spectroscopy (XPS) analyses [45].

## 4. Final Discussion on the Role of Alumino-Silicate and Carbonate Mineral Fines in the Alkali-Activation Process

The extensive literature review performed in this paper allowed the ascription of a positive role played by both alumino-silicate and carbonate mineral fines on the alkali activation process.

In the case of alumino-silicate waste, a positive effect on limiting crack formation due to drying shrinkage [47] and on decreasing the brittleness of quickly reacted geopolymer matrix [47] is widely recognized. The volumetric stability is maintained during high-temperature exposures (up to 800 °C), and, therefore, alumino-silicate mineral-added geopolymers show an improved behavior for high-temperature applications.

A positive role is generally recognized for carbonate fines, too. In fact, their addition improves the mechanical properties, limits the shrinkage effects, and decreases the material porosity. However, the potential of this mineral to increase the high-temperature stability of alkali-activated materials is compromised by the carbonates’ thermal decomposition, starting from about 700 °C.

In addition to their role as fillers, several authors claim an ‘active’ role in the alkali activation processes, even if crystalline alumino-silicates and carbonates show a low solubility within alkaline solutions.

**Figure 11:**
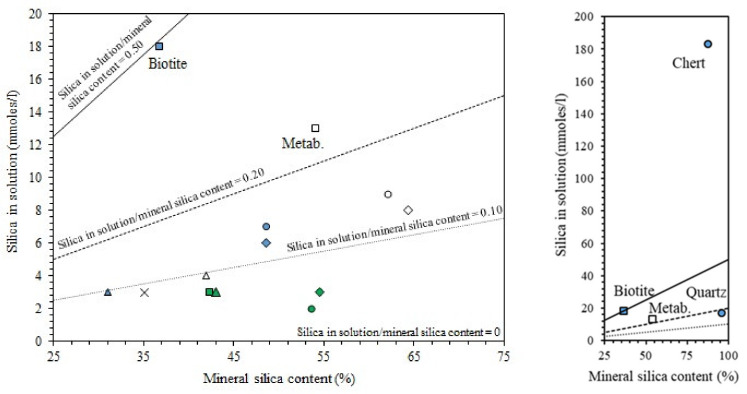
Silica in solution as a function of silica content of silicate minerals [103].

A comprehensive study of the solubility of common silicate and carbonate minerals under strong alkaline conditions was performed by Choquette et al. [103]. While carbonates (calcite, and especially dolomites) show a strong corrosion in 1 M NaOH solution at 23 °C, silicate dissolution needs a thermal activation (80 °C for 24 h in 1 M NaOH solution). Under such conditions, the behavior of different silica species was classified into high-, moderate-, and low-reactivity areas (Figure 11). Chert showed the highest solubility, which was one order of magnitude higher than all the other species. It was followed by biotite (placed at the boundary between high- and moderate-solubility regions), then by metabentonite, quartz, and feldspars.

This study can be exploited as a useful tool to select minerals with a certain potential in the alkali activation process. It is worth mentioning that the only work related to the alkali activation of pure, untreated alumino-silicates exploits the high/moderate reactivity of biotite, quartz, and feldspars phases contained in waste granite fines [46]. Samples were cured at 80 °C for 48 h, which allowed dissolution of these species, according to [103]. Dissolved species from the particles’ surfaces contribute to the formation of an alumino-silicate gel, which binds the unreacted particles, acting as a matrix reinforcement.

Anyway, for both alumino-silicates and carbonate waste, a key role for the reactivity is played by particle fineness [13,72,104]; the lower the size, the higher the reactivity and the final mechanical properties. At the same time, the alkaline solution plays an important role in the alkali activation process [105,106], and a very alkaline solution strongly influences particle dissolution, whether their nature is siliceous [51,52] or carbonatic [58,66].

In the case of carbonate mineral addition, most of the authors agree in claiming that the achievement of the optimal addition amount is the key for optimizing the mechanical properties. In fact, on one side, several positive roles have been assigned to carbonate fines during the alkali activation process: (i) dissolution under alkaline medium to form Ca(OH)_2_ and subsequent carbonation to produce calcite [55]; (ii) reaction with silica to form C-S-H gel [55]; (iii) acceleration of setting and hardening stages, acting as a nucleation site and promoting reactions and gel formation [65,69,70]; (iv) improvement of leaching behavior of Al and Si ions from alumino-silicate sources [67]; (v) filler action able to reduce the total porosity [68], and generation of strong interfaces with the matrix [62]. On the other hand, all of these mechanisms seem to be active until a certain carbonate amount, while further additions induce an opposite trend in the mechanical property development due to a ‘dilution’ effect of the reactive matrix, and, precisely, the lack of silica and alumina necessary for geopolymer network formation [66].

It should be mentioned that this optimal value strongly depends on the raw materials and processing conditions used; values range from 10 to 50 wt.% in MK [55,57,67,71], from 10% to 40% in GBFS [69,70,72], and up to 60% in FA [66].

However, also in this case, a few examples [40,45] demonstrated that it is possible to exploit pure carbonate fines to produce alkali-activated materials with satisfying mechanical properties. In both cases, an active role of Ca^2+^ ions in reacting with the activating solution was proved, providing C-S-H species and a condensed gel network surrounding the unreactive particles, with resulting high mechanical properties.

Finally, the study by Choquette et al. [103] showing the occurrence of a certain dissolution of both alumino-silicate and carbonate minerals under alkaline conditions suggests the possible synergic exploitation of these fines in the alkali activation process.

**Figure 12:**
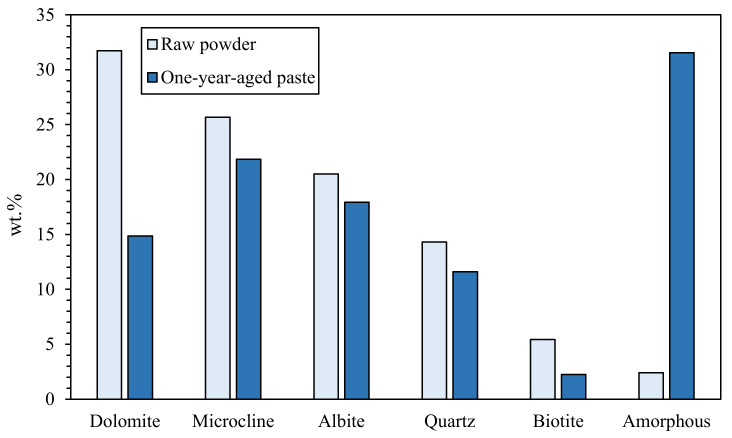
X-ray diffraction (XRD) phase quantification of as-received mud (containing both granite and dolomite waste fine) and of one-year-aged sample. Redrawn from [46] under the license n. 4792980067658.

Accordingly, in Table 1, some examples of mixed alumino-silicate/carbonate systems are collected. With specific reference to Reference [46], in which granite mud was ‘polluted’ by dolomite waste and this mixture was used to prepare alkali-activated materials, XRD performed on fresh and one-year-aged samples showed the effective dissolution of both alumino-silicates and dolomite phases, thus suggesting their synergic role in increasing the performance of the hardened materials (Figure 12).

A summary of the previous authors’ studies concerning the development of alkali-activated materials obtained by ‘pure’ mineral wastes is depicted in Table 4. In particular, the behaviors of a pure granite powder [46] and a pure marble mud [45] are compared to those of mixed raw materials: A granite mud containing ~30 wt.% of dolomite [46], and a marble mud contained 4.6 wt.% of waste glass fines [45]. First, it can be observed that both pure granite and marble muds were characterized by high mechanical properties (compressive strength in the range 30–35 MPa and flexural strength > 11 MPa). The mixed granite–dolomite mud presented very close mechanical values. However, a poor behavior of the 100% granite sample was shown under immersion in water, leading to a rapid disintegration of the samples. Conversely, a significantly improved resistance was observed for the 100% carbonate and the mixed 70/30 granite–carbonate material due to the strengthening role provided by C-S-H species. In fact, no disintegration of the samples occurred, even if the formation of some cracks on the samples’ surface was observed. Hydraulic stability of geopolymers is highly debated in the literature. Several authors reported a decrease of mechanical properties or even disintegration of Ca-free samples due to water immersion [107,108,109,110,111,112]. This issue can be associated with the high solubility of excess, unreacted alkaline solution, providing ineffective binding properties under water and/or to the weak bonds occurring in improperly cured geopolymers [108,109,113,114]. Conversely, in high-Ca-content materials, the formation of C-S-H containing gels provides a key effect for withstanding water immersion [45,46,51,115,116]. As a last attempt, the marble mud was added with finely milled glass powder as a readily available amorphous silica source [45]. A positive effect was stated, both in mechanical properties, which were superior to those of the other mineral-derived pastes, and, above all, on the water resistance, which was further improved, as no cracks appeared in these materials, probably due to the formation of more C-S-H species.

These results pave the way towards the full exploitation of mineral waste in the alkali activation process, even if, in the future, further studies need to be carried out to finalize the composition and to deepen all of the durability aspects.

## 5. Conclusions

This review examined the current state of the art relevant to the development of alkali-activated materials using natural stone waste and minerals. It reported on the progress achieved by the scientific community in this field by exploring the effect of either alumino-silicate or carbonate waste powder additions (or a combination of both) on the workability of the fresh mixtures and on the mechanical and durability properties of the hardened materials as a function of raw material composition, alkaline activators, and curing conditions. The role of alumino-silicate and carbonate mineral fines in the alkali activation process was discussed. In particular, a positive effect of alumino-silicate fines in the development of alkali-activated materials was reported due to their ability to reduce shrinkage crack formation and brittleness, as widely recognized in numerous studies. In addition, the volumetric stability under high temperature exposures demonstrated by the hardened materials obtained through alkali activation with addition of alumino-silicate waste confirms the positive role of these minerals and their potentiality for a wide range of applications, also in extreme conditions. A positive effect was generally reported for carbonate fines, too, by improving the mechanical properties, limiting shrinkage, and decreasing the material porosity, with a major limitation given by the carbonates’ thermal decomposition under high temperature exposures. Apart from the beneficial filler effect provided by stone waste additions, as demonstrated in a variety of studies, an ‘active’ role in the alkali activation process was also attributed by some authors to alumino-silicate and carbonate fines, despite their low solubility within alkaline solutions. Curing temperature, particle fineness, and activating solution pH were recognized as the main parameters affecting the dissolution and polymerization reactions, and hence the final mechanical properties. The determination of the optimal proportion of stone waste to be added in order to maximize the hardened material properties is still an unsolved matter, which needs further investigation to be fully understood. However, promising results were obtained in a few studies, where either alumino-silicate or carbonate stone waste were used not as a partial replacement, but as the main active ingredient in the alkali activation process, thus opening new perspectives for the reuse of these materials as a sustainable alternative to their landfill disposal.

## Figures and Tables

**Table 1:** Summary of the papers related to the use of stone waste/minerals in alkali activation processes. A classification of the minerals based on their chemical nature (alumino-silicate or carbonate) is provided, in addition to the composition of the ‘active’ raw materials for alkali activation (if any) and the general composition of the liquid alkaline activator.

Classification	Mineral Additive	Alkali-Activation Source	Activator	Reference
Alumino-silicates	Granite	Fly Ash/Granulated Blast Furnace Slag	NaOH + Na_2_SiO_3_ + H_2_O	[47]
	Granite	Metakaolin	NaOH + Na_2_SiO_3_ + H_2_O	[48]
	Granite	-	NaOH + Na_2_SiO_3_ + H_2_O	[46]
	Albite ^+^	-	Na_2_CO_3_/NaOH	[49]
	Pietra Serena	Metakaolin	NaOH + Na_2_SiO_3_ + H_2_O	[50]
	Pisha sandstone	-/Fly Ash	NaOH + Na_2_SiO_3_ + H_2_O	[51]
	Pisha sandstone	-	NaOH + NaCO_3_ + Na_2_SO_4_ + Na_2_SiO_3_ + H_2_O	[52]
	Cordierite	Metakaolin	NaOH + Na_2_SiO_3_ + H_2_O	[53]
	Diatomite	-	NaOH + H_2_O + Wood Biomass Ash ^	[54]
Carbonates	Calcite	Metakaolin	NaOH + Na_2_SiO_3_ + H_2_O	[55]
	Calcite	Granulated Blast Furnace Slag	NaOH + H_2_O	[56]
	Calcite	Metakaolin	NaOH/KOH + Na_2_SiO_3_ + H_2_O	[57]
	Calcite	Fly Ash	NaOH + Na_2_SiO_3_ + H_2_O	[58]
	Dolomite	Fly Ash/Granulated Blast Furnace Slag	Na_2_CO_3_/NaOH	[59]
	Dolomite	Metakaolin	NaOH + Na_2_SiO_3_ + H_2_O	[55]
	Dolomite ^+^	Bentonite + Na_2_CO_3_	H_2_O	[60]
	Dolomite ^+^	Granulated Blast Furnace Slag + Na_2_CO_3_	H_2_O	[61]
	Dolomite	Fly ash/cement	NaOH + Na_2_SiO_3_ + H_2_O	[62]
	Marble °	-	NaOH + H_2_O	[63]
	Marble	-	NaOH + Na_2_SiO_3_ + H_2_O	[45]
	Marble	Smectite clay	NaOH + Na_2_SiO_3_/Sodium citrate + H_2_O	[64]
	Marble	Cement/Fly ash/GBFS/Gypsum/Clay	NaOH + Na_2_SiO_3_ + H_2_O	[65]
	Marble	Fly ash	NaOH + Na_2_SiO_3_ + H_2_O	[66]
	Pietra di Angera	Metakaolin	NaOH + Na_2_SiO_3_ + H_2_O	[50]
	Travertine °	-	NaOH + H_2_O	[63]
	Limestone	Metakaolin	NaOH + H_2_O	[67]
	Limestone	Fly Ash/Granulated Blast Furnace Slag	NaOH + Na_2_SiO_3_ + H_2_O	[68]
	Limestone	Granulated Blast Furnace Slag	Na_2_CO_3_ + H_2_O	[69,70]
	Limestone	-	NaOH + Na_2_SiO_3_ + H_2_O	[40]
	Limestone	Halloysite clay	NaOH + Na_2_SiO_3_+H_2_O	[71]
	Limestone *	Granulated Blast Furnace Slag	NaOH + Na_2_CO_3_ + H_2_O	[72]
Mixed	Dolomite + Microcline + Albite + Quartz	-	NaOH + Na_2_SiO_3_ + H_2_O	[46]
	Marl ^+^	-	NaOH + Na_2_SiO_3_ + H_2_O	[73]
	Marl ^+^+Limestone	-	Na_2_SiO_3_ + H_2_O	[74]
	Marl/Marl ^+^	Granulated Blast Furnace Slag	Na_2_SiO_3_ + H_2_O	[75]
	Pietra serena sludge	Fly ash/metakaolin	NaOH + H_2_O	[76]
	Garnet tailings	Metakaolin	Na_2_SiO_3_ + NaOH + H_2_O	[77]

* Calcite + Dolomite/Calcite/Calcite + Quartz. ° Marble and Travertine were used in combination with Tuff. ^+^ Calcined. ^ Mainly calcium carbonate.

**Table 2:** Chemical composition according to X-ray fluorescence spectroscopy analyses (XRF), alkali-activating solution details, curing conditions, and compressive strength of alkali-activated materials containing alumino-silicate minerals.

Mineral Additive (Chemical Composition)	Alkali Activation and Curing Regimen	Max. Compressive Strength ^+^	Reference
Granite (62.11% SiO_2_; 15.72% Al_2_O_3_; 4.98% K_2_O)	10 M NaOH	30.5 MPa (10% of granite in FA-based geopolymers); 72.6 MPa (10% of granite in GBFS-based geopolymers)	[47]
	80 °C for 24 h		
Granite (68.10% SiO_2_; 15.80% Al_2_O_3_; 5.32% K_2_O)	M_s_ * = 1.64 (H_2_O/Na_2_O molar ratio of 13)	35 MPa	[46]
	80 °C for 24 h		
Pietra Serena (59% SiO_2_; 16% Al_2_O_3_; 6.3% MgO)	10, 14, 16 and 20 H_2_O/Na_2_O molar ratio (mixing H_2_O + Na_2_SiO_3_ + NaOH)	21 MPa (H_2_O/Na_2_O molar ratio = 20; metakaolin:pietra Serena = 1:1)	[50]
	20 °C and 90% RH		
Granite (60.51% SiO_2_; 17.49% Al_2_O_3_; 8.71% Fe_2_O_3_)	Na_2_SiO_3_ was used to activate fused granite (with several M_s_^*^) and MK (added to balance Na_2_/Al_2_O_3_ ratio)	40.5 MPa (for mortars containing fused granite wastes with SiO_2_/Na_2_O = 0.47 and Al_2_O_3_/Na_2_O = 0.08)	[48]
	24 h at room temperature closed in plastic bags, then 25 °C and 90% RH		
Pisha sandstone (65.64% SiO_2_; 14.35% Al_2_O_3_; 8.02% CaO)	0.7, 1.2, and 1.6 wt.% of NaOH with respect to 100 g of Pisha sandstone	6 MPa (ambient cured and 1.2 wt.% NaOH)	[51]
	80 °C for 24 h then ambient and water immersed		
Pisha sandstone (62.46% SiO_2_; 20.08% Al_2_O_3_; 5.10% CaO)	Na_2_SiO_3_ (M_s_^*^ = 1.5, 2.0 and 3.0); Na_2_CO_3_, Na_2_SO_4_; NaOH	14.4 MPa (Na_2_SiO_3_ with M_s_ = 3, cured at 80 °C and milled Pisha stone)	[52]
	(i) 80 °C for 24 h and (ii) ambient temperature		
Cordierite (52.85% SiO_2_; 34.62% Al_2_O_3_; 11.66% MgO)	H_2_O/Na_2_O = 13, 15, and 20	57.5 MPa (30% of Cordierite; H_2_O/Na_2_O = 13)	[53]
	sealed and room temperature		
Diatomite (80.3% SiO_2_; 6.1% Al_2_O_3_; 6.79% Fe_2_O_3_)	3 wt.% of NaOH; w/p = 0.27	48 MPa	[54]
	23 °C and 99% RH		
Albite (70.9% SiO_2_; 17% Al_2_O_3_; 9.75% Na_2_O)	Albite calcined with NaOH or Na_2_CO_3_; w/s = 0.3	44.2 MPa (Albite calcined with 50% NaOH at 1000 °C)	[49]
	sealed and room temperature		

^+^ 28 days. * Ms = SiO_2_/Na_2_O molar ratio.

**Table 3:** Chemical composition according to X-ray fluorescence spectroscopy analyses (XRF), alkali-activating solution details, curing conditions, and compressive strength of alkali-activated materials containing carbonate minerals.

Mineral Additive (Chemical Composition)	Alkali-Activation and Curing Regimen	Max. Compressive Strength ^+^	Reference
Calcite (53.5% CaO; 1.7% MgO; 1.5% SiO_2_)	M_s_^*^ = 2.0, 1.5, and 1.2	~60 MPa (M_s_ = 1.5, MK with 20% of calcite)	[55]
	40 °C for 24 h		
Calcite (55.91% CaO; 0.18% K_2_O; 0.09% SiO_2_)	2%, 4%, and 6% of NaOH	~70 MPa (4% of NaOH, 5% of calcite, and 91% of GBFS)	[56]
	37 °C 100% RH		
Calcite (purity 98.5%)	13% KOH, 10% NaOH, 27% Na_2_SiO_3_, 50% H_2_O	~28 MPa (MK with 6% of calcite)	[57]
	40 °C for 12 h		
Calcite (50.1% CaO; 3.9% SiO_2_; 1.7% Al_2_O_3_)	3, 6, and 12 M NaOH	19.2 MPa (12 M, 67% mineral addition, 5 h curing)	[58]
	80 °C for 1, 3, and 5 h		
Marble (53.68% CaO; 1.32% Fe_2_O_3_; 0.26% SiO_2_)	1, 5, and 10 M NaOH	37.48 MPa (10 M NaOH, curing 20 °C, 20% Marble)	[63]
	(1) 22 °C and 40% RH; (2) 45 °C for 24 h; (3) 75 °C for 24 h. Afterwards, wet condition (min. 95% RH) or 22 °C and 35% RH		
Marble (55.9% CaO; 0.6% MgO; 0.1% Fe_2_O_3_)	(1) 0.1 mol of Na_2_O; (2) 0.1 mol of Na_2_O and 0.1 mol SiO_2_; (3) substitution of sodium citrate (Na_3_C_6_H_5_O_7_)	60.7 MPa (25% Marble/75% calcined smectite clay; containing Na-citrate)	[64]
	24 h at 95% RH then dry-cured at room temperature		
Marble (44.20% CaO; 1.41% MgO; 0.07% Fe_2_O_3_)	M_s_^*^ = 1.65 and 3.50	38.30 MPa (M_s_ = 1.65, dry curing)	[45]
	24 h at 80 °C then: (1) 20 °C and 95% RH; (2) 20 °C and 18% RH; (3) immersed in water		
Marble (45.60% CaO; 6.82% MgO; 0.70% SiO_2_)	8 M NaOH or Na_2_SiO_3_·nH_2_O:NaOH (w:w = 5)	52 MPa (Na_2_SiO_3_·nH_2_O + NaOH; cement, GBFS, marble, FA)	[65]
	Room temperature		
Marble (38.02% CaO; 34.66% SiO_2_; 13.12% Fe_2_O_3_; 7.21% MgO)	2 and 4 M NaOH + Na_2_SiO_3_	6.52 MPa (7 days, 4 M, 60% marble + 40% FA)	[66]
	70 °C for 24 h and 7 days in plastic bags		
Travertine (55.10% CaO; 0.70% SiO_2_; 0.20% Fe_2_O_3_)	1, 5, and 10 M NaOH	42.24 MPa (10 M NaOH, dry curing at 20 °C, 20% travertine)	[63]
	(1) 22 °C and 40% RH; (2) 45 °C for 24 h; (3) 75 °C for 24 h. Afterwards, wet condition (min. 95% RH) or 22 °C and 35% RH		
Dolomite (33.4% CaO; 17.1% MgO; 2.5% SiO_2_)	M_s_^*^ = 2.0, 1.5, and 1.2	~45 MPa (M_s_ = 1.5, MK with 20% of dolomite)	[55]
	40 °C for 24 h		
Dolomite (42.48% CaO, 19.15% MgO)	M_s_^*^ = 2.5 (Na_2_CO_3_ and bentonite)	38.3 MPa (bentonite, dolomite and Na_2_CO_3_ calcined at 1110 °C and (CaO + MgO)/SiO_2_ = 2.1)	[60]
	80 °C for 3 days		
Dolomite (74.8% CaO; 18.3% MgO; 3.7% SiO_2_)	M_s_^*^= 0.93 (10 M NaOH + Na_2_SiO_3_)	~60 MPa (Cement with 40% of dolomite); ~40 MPa (FA with 40% of dolomite);	[62]
	Cement-based samples 24 h at 100% RH then immersed in lime water; FA-based samples 24 h at 40 °C and 100 % RH then sealed and at room temperature		
Dolomite (31.4% CaO, 21.3% MgO, 1.1% SiO_2_)	Na_2_CO_3_ + calcined dolomite (Na_2_O = 4.9%–7.6% in the dry mixture)	41.6 MPa (GBFS with 10% of calcined dolomite and 10% of Na_2_CO_3_)	[61]
	20 °C and RH > 95% ± 2%		
Dolomite (27.13% CaO; 24.53% MgO; 0.13% SiO_2_)	4 M NaOH or 2 M Na_2_CO_3_	~60 MPa (NaOH, GBFS and 20% of dolomite); ~80 MPa (Na_2_CO_3_, GBFS and 20% of dolomite);	[59]
	20 °C 100% RH		
Pietra di Angera (64% CaO; 33% MgO; 2.2% Fe_2_O_3_)	10, 14, 16, and 20 H_2_O/Na_2_O molar ratio (obtained mixing H_2_O + Na_2_SiO_3_ + NaOH)	18 MPa (H_2_O/Na_2_O molar ratio = 20; MK:pietra di Angera = 1:1)	[50]
	20 °C and 90% RH		
Limestone (53.5% CaO; 1.7% MgO; 1.5% SiO_2_)	3 and 5 M NaOH	7 MPa (50% LM/50% MK; 5 M NaOH; 20 °C; dry curing)	[67]
	24 h at 20 or 80 °C then wet curing (water immersed) or dry curing (laboratory conditions)		
Limestone (53.96% CaO; 1.01% MgO; 0.84% SiO_2_)	M_s_ * = 1.4	83.5 MPa (30% LM/10% FA/60% GBFS)	[68]
	20 °C and 95% RH		
Limestone (53.96% CaO; 1.01% MgO; 0.84% SiO_2_)	4 wt.% of Na_2_O (starting from Na_2_CO_3_)	~55 MPa (GBFS with 10% of LM)	[69,70]
	20 °C and 95% RH		
Limestone (57.43% CaO; 1.06% SiO_2_)	M_s_ * = 0 (only NaOH), 1, and 1.5	~15 MPa (M_s_ = 1, 10% Na_2_O)	[40]
	24 h at 60 °C then dry stored in plastic bags at 20 °C and 80%–90% RH		
Limestone (47.85% CaO; 9.07% SiO_2_; 1.51% Al_2_O_3_)	5, 10, and 8 M NaOH + Na_2_SiO_3_	47.77 MPa (8 M, 45% LM/55% Clay)	[71]
	24 °C in open air		
Limestone 1 (90% calcite, 9% quartz) (43.31% CaO; 14.26% SiO_2_; 2.44% Al_2_O_3_)	NaOH + Na_2_CO_3_ + H_2_O	39 MPa (30% LM_3/70% GBFS; S_sp_ 600 m^2^/kg)	[72]
Limestone 2 (33% calcite, 66% dolomite) (39.79% CaO; 1.26% SiO_2_; 12.94% MgO)			
Limestone 3 (100% calcite) (55.06% CaO; 0.47% SiO_2_; 0.49% MgO)	Room temperature and 95%–100% RH		

^+^ 28 days. * M_s_ = SiO_2_/Na_2_O molar ratio.

**Table 4:** Compositions and properties of pastes derived from the alkali activation of pure and mixed alumino-silicate and carbonate muds [45,46].

	Pure Alumino-Silicate	Mixed Alumino-Silicate/Carbonate	Mixed Carbonate/Silica	Pure Carbonate
Composition	100% granite	~70% granite	95.4% marble	100% marble
		~30% dolomite	4.6% waste glass	
28 day compressive strength	~30 MPa	~30 MPa	~45 MPa	>35 MPa
28 day flexural strength	~12 MPa	~14 MPa	~17 MPa	~11 MPa
Water resistance	Rapid dissolution	Crack formation	Optimal behavior	Crack formation

## Abbreviations

^29^Si MAS-NMR	^29^Si Magic Angle Spinning Nuclear Magnetic Resonance
C-A-H	Calcium Aluminate Hydrates
C-A-S-H	Calcium Aluminum Silicate Hydrate
N-A-S-H	Sodium Aluminum Silicate Hydrate
C-S-H	Calcium Silicate Hydrates
TGA/DTG	Thermo-Gravimetric Analysis coupled with Derivative Thermo-Gravimetric Analysis
EDX	Energy Dispersive X-ray Spectroscopy
FA	Fly Ash
FESEM	Field Emission Scanning Electron Microscopy
FT-IR	Fourier-Transform Infrared Spectroscopy
GBFS	Granulated Blast Furnace Slag
LS	Limestone
MK	Metakaolin
Ms	SiO_2_/Na_2_O molar ratio
RH	Relative Humidity
SEM	Scanning Electron Microscopy
XPS	X-ray Photoelectron Spectroscopy
XRD	X-ray Diffraction
XRF	X-ray Fluorescence Spectroscopy
WBA	Wood Biomass Ash
